# Advanced medical micro-robotics for early diagnosis and therapeutic interventions

**DOI:** 10.3389/frobt.2022.1086043

**Published:** 2023-01-10

**Authors:** Dandan Zhang, Thomas E. Gorochowski, Lucia Marucci, Hyun-Taek Lee, Bruno Gil, Bing Li, Sabine Hauert, Eric Yeatman

**Affiliations:** ^1^ Department of Engineering Mathematics, University of Bristol, Bristol, United Kingdom; ^2^ Bristol Robotics Laboratory, Bristol, United Kingdom; ^3^ School of Biological Sciences, University of Bristol, Bristol, United Kingdom; ^4^ BrisEngBio, University of Bristol, Bristol, United Kingdom; ^5^ Department of Mechanical Engineering, Inha University, Incheon, South Korea; ^6^ Department of Electrical and Electronic Engineering, Imperial College London, London, United Kingdom; ^7^ The Institute for Materials Discovery, University College London, London, United Kingdom; ^8^ Department of Brain Science, Imperial College London, London, United Kingdom; ^9^ Care Research & Technology Centre, UK Dementia Research Institute, Imperial College London, London, United Kingdom

**Keywords:** micro-robotics, medicine, biomedical engineering, Artificial Intelligence, micro-manipulation

## Abstract

Recent technological advances in micro-robotics have demonstrated their immense potential for biomedical applications. Emerging micro-robots have versatile sensing systems, flexible locomotion and dexterous manipulation capabilities that can significantly contribute to the healthcare system. Despite the appreciated and tangible benefits of medical micro-robotics, many challenges still remain. Here, we review the major challenges, current trends and significant achievements for developing versatile and intelligent micro-robotics with a focus on applications in early diagnosis and therapeutic interventions. We also consider some recent emerging micro-robotic technologies that employ synthetic biology to support a new generation of living micro-robots. We expect to inspire future development of micro-robots toward clinical translation by identifying the roadblocks that need to be overcome.

## 1 Introduction

Our growing ability to control the microscopic world has led to a surge in robotics research, with a focus on small-scale systems (Dabbagh et al., 2022). Small-scale robotics cover nano-robotics, micro-robotics and milli-robotics according to their characteristic dimensions. Here, we focus on micro-robotics with characteristic dimension between a millimeter and micrometer, where mechanics are dominated by micro-scale forces and physical phenomena.

A fundamental challenge for designing micro-robots is that the miniaturization of larger robotic designs is not always feasible, since the physical properties of macro and micro environments differ significantly. For example, the surface-to-volume ratio increases at the micro-scale causing surface properties and forces to dominate (Chen et al., 2010a). Other practical challenges also hamper the development of robots at the micro-scale. For example, fabrication, actuation, sensing, and energy supply all require new approaches. *In vivo* applications add further complications, with safety and biocompatibility being crucial for effective clinical translation.

**FIGURE 1:**
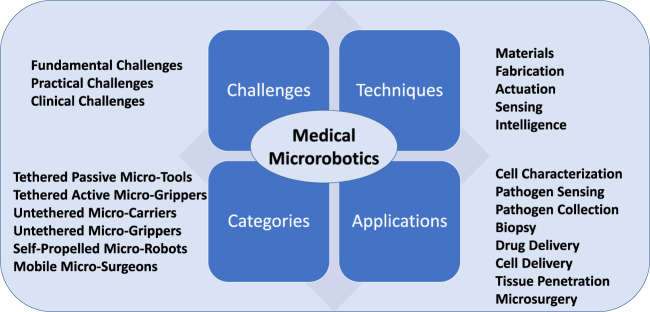
Conceptual overview of this article. Key challenges, techniques, development tendencies and applications of micro-robotics for biomedical research are shown.

Recent advances in micro-fabrication, nanotechnology, and materials engineering have addressed some of the challenges mentioned above, and enabled micro-robotics to be developed for a variety of biomedical applications Dabbagh et al. (2022). These include single-cell investigation (Grexa et al., 2020), biopsy (Jin et al., 2020), targeted drug delivery (Jang et al., 2019), regenerative medicine (Li et al., 2018), clearing of clogged blood vessels (Hosney et al., 2016), and microsurgery (Děkanovskỳ et al., 2022). For *in vitro* biomedical applications, micro-robots can enhance the precision and repeatability of manipulation at the micro-scale and reduce the workload of a human. For *in vivo* applications, micro-robots can enhance the dexterity of manipulation, allow surgeons access to hard-to-reach anatomy, and perform delicate interactions with tissues to reduce the invasiveness of a procedure. We will summarize typical biomedical applications in this review, including tasks related to diagnosis (e.g., sensing, monitoring, and tracking of pathogens) and therapeutic interventions (e.g., biopsy, drug/cell delivery, cancer therapy, and microsurgery). The overview of this article is shown in Figure 1.

The term “robot” normally refers to a machine that can perform complex tasks automatically, while “micro-robot” indicates the robot is sub millimeter in size. However, the term micro-robot has been used to describe micro-motors, micro-swimmers, micro-tools, micro-carriers, micro-grippers, and micro-machines in existing literature as well. In this review, we consider micro-robots to cover all the aforementioned terms. We begin this review by illustrating the challenges of developing micro-robots in different aspects. We then discuss the emerging technologies that help overcome some of these hurdles to enable the construction and integration of micro-robotic systems. Following that, we categorize the types of micro-robot available today and provide a rigorous classification of micro-robots based on their abilities to be tethered or untethered, passive or active, remotely-actuated or self-propelled. Then we explore applications of medical micro-robots for early diagnosis and therapeutic interventions. Finally, we provide an outlook on the field and the possibility to engineer biology itself as a means to implement living micro-robots.

## 2 Challenges

In this section, we identify the challenges for micro-robotics research in three aspects, including fundamental challenges, practical challenges, and clinical challenges.

### 2.1 Fundamental challenges

At the micro-scale, volumetric effects such as buoyancy, gravity, and inertial forces are almost negligible. Instead, surface tension, adhesion, fluid viscosity, friction, Van der Waals forces, electrostatic and capillary forces dominate (Fearing, 1995). Van der Waals forces are always present at the micro-scale, while other forces become dominant according to the materials used to fabricate the micro-robots and the environment (Setti, 2007).

The major differences seen for the dominating effects mean that physical models developed for the control of large-scale robotics often do not apply when scaled-down to micro-robotic systems. Non-linearity brought about by the physical properties of the micro-scale world make precise, reliable, and flexible manipulation of biological micro-objects by micro-robots challenging, since micro-manipulation may be influenced by the size, geometry, and material type of the targeted objects. Furthermore, micro-robotic systems are sensitive to environmental parameters such as temperature, humidity and surface chemistry. Adding to these difficulties, in medical applications, micro-robots used for *in vivo* tasks must be able to handle complex environments with changing physiological conditions (e.g., soft tissue vs. blood) and unanticipated biological events. The physical models for the design of micro-robotic systems in these contexts must therefore be modified according to specific conditions under which the system will be exposed.

### 2.2 Practical challenges

Beyond fundamental challenges that affect all micro-scale systems, there are also practical challenges associated specifically with the construction of micro-robotic systems. For example, reliable actuation, sensing systems and control systems are required for efficient manipulation of biological micro-objects. For micro-robots, all on-board components such as actuators, sensors, power supplies, and controllers must be miniaturized to the micron scale, which is often difficult with conventional fabrication techniques and approaches to these aspects of robot design. The exploration of new manufacturing techniques and effective control and actuation methods require further research with careful consideration of the cost and ability to handle multi-material processes for each of these areas.

To realize the full potential of micro-robots, there is a strong need for new micro-fabrication techniques, in conjunction with innovative power and energy system design, that will likely lean on novel structural design methods and advanced material development (Liu et al., 2015). Because the overall dimensions of micro-robots are too small to integrate commonly used electronic circuits, conventional strategies, such as electronic computing circuitry and software cannot be directly translated to micro-robots. New strategies are required to tackle these challenges in the assembly of advanced micro-robotics through materials, novel fabrication methods, integrated actuation and sensing capacities based on new mechanisms.

Outside of micro-robot construction, deployment of these systems for micro-manipulation applications offer yet more challenges. Due to adhesion forces, micro-objects tend to stick to micro-grippers or the substrates of the micro-robot itself. A large body of research has aimed to solve this issue by utilizing dynamical effects (Beyeler et al., 2006), gluing (Grutzeck, 2005), mechanical release (Bark et al., 2002), and gas injection (Haliyo et al., 2003). New techniques have also been developing to change the contact angle between the liquid bridge and the gripper surface *via* electrowetting (Vasudev and Zhe, 2008). New designs have also been proposed to reduce the contact surface between the micro-objects and the end-effectors of micro-robots by optimizing micro-gripper shape (Biganzoli et al., 2005).

### 2.3 Clinical challenges

Further challenges come from transferring micro-robotic systems from laboratory to clinical settings (Ongaro et al., 2016a). Laboratory-based micro-robotic techniques are increasingly mature, since most of the experiments were carried out in a homogeneous, Newtonian environment, under optical microscopes. However, for clinical practice, many other challenges need to be solved.

Micro-robots have the potential to target cancer tissues more directly and could be engineered to embed sensing and decision-making capabilities (Schmidt et al., 2020). Micro-robots for cancer targeting can broadly be classified as biologically actuated, synthetic, or hybrid if composed of cellular, synthetic, or mixed components, respectively (Gao and Wang, 2014; Thanuja et al., 2018). In all cases, active motion can be leveraged to obtain penetration and accumulation at tumour sites. Micro-robots can also be used to deliver drugs (not just in tumor tissues) in which case different mechanisms are used (e.g., chemical or enzymatic based) to control the timing and correct destination (Liu et al., 2022). This is different from *in vitro* applications where microscopic data can be accessed with ease. At present, most micro-robots are evaluated based on *in vitro* studies, while the *in vivo* applications are challenging.

For *in vivo* applications, the trackability of micro-robots with medical imaging devices become another major hurdle Aziz et al. (2020). Therefore, advanced medical imaging techniques are worth developing to track and localize micro-robots Pané et al. (2019). Biocompatibility and biodegradability have to be considered when selecting the materials to fabricate micro-robots to be used in the body Wang et al. (2018). Furthermore, how to retrieve degraded or malfunctioning micro-robots when they have conducted micro-surgical tasks is an open challenge (Ceylan et al., 2019). The development of retrievable Iacovacci et al. (2018) micro-robots is required.

For *in vitro* applications, micro-robots normally navigate in homogenous Newtonian fluid-like environments. However, for *in vivo* applications, micro-robots need to work in complex media Wu et al. (2020). For example, micro-robots commonly need to travel against blood flows, whose fluidic and physiological characteristics are non-Newtonian. Rheological properties of non-Newtonian biological fluids may limit the propulsion of micro-robots using traditional actuation techniques (Aghakhani et al., 2022). Moreover, the pH levels of the physiological environment may influence the performance of the micro-robots. Therefore, proper materials should be selected to avoid degradation in acidic environments. In addition, biological barriers, such as tight junctions and flow/rheological barriers, are known to act as obstacles to mobility (Ceylan et al., 2019). These obstaces are typically located in the entry points and may prevent micro-robots from reaching their full potential (Stillman et al.,2020).

Another challenge that micro-robots encounter during clinical translation is the host immune system and the issue of triggering innate defense mechanisms. To solve this issue, Zwitterionic materials have been used to develop non-immunogenic stealth micro-robots that avoid detection and phagocytosis from macrophages (Cabanach et al., 2020). Biohybrid micro-robots might offer good solutions to toxicity issues, while enlarging the set of possible functionalities. Structural parameters, surface chemistry and morphology of micro-robots can also be optimized to reduce the immune response by minimizing the physical interactions with the immune system (Yasa et al., 2020). In addition, micro-robots could be designed to enhance the immune system by promoting immunotherapy.

Ethical questions and risks should also be assessed before using micro-robots to act on certain organs of the human body directly. Moreover, to pass regulatory hurdles, safety and efficacy of using micro-robots must be demonstrated (Knoepfler, 2015; Agrahari et al. (2020).

## 3 Key technologies

**FIGURE 2:**
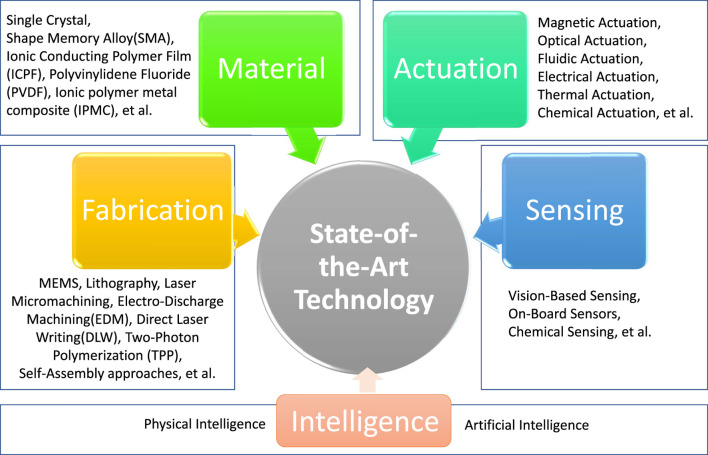
Technologies required for the development of medical micro-robots.

Figure 2 shows relevant research areas that must be considered for the development of advanced micro-robots. In this section, we will explore the key techniques in depth covering developments to date, as well as remaining hurdles.

### 3.1 Materials for micro-robotics

Many different types of material have been assessed for the construction of micro-robotics. The materials used for the construction of micro-robotics can be categorized into i) catalytic and transient materials, ii) responsive materials, and iii) hybrid materials which combine various types of power sources.

Catalytic and transient materials include noble metals (Pt) (Lyu et al., 2021), transient metals (iron) (Karshalev et al., 2017), minerals (calcium carbonate) (Guix et al., 2016), metal alloys, semiconductors, and enzymes (Patino et al., 2018). These materials allow the gathering of chemical energy from the surrounding solutions and convert them into diverse motions. Responsive materials include magnetic materials (Zhang et al., 2019), photothermal and photocatalytic materials (Dong et al., 2018), and polarizable materials. These materials respond to the external field change or external parameters (such as temperature), which generate the mechanical force for motions (Chang et al., 2007). Hybrid materials, developed based on the combination of various power sources, take advantage of both local and external power sources as the driving force. For example, with a combination of chemical and magnetic materials, the micro-robot can convert both chemical energy and magnetic field into their driving forces (Gao et al., 2011). Here, we briefly introduce some of the most advanced and popular materials for the fabrication of micro-robotics. For example, polyvinylidene fluoride (PVDF) exhibits strong piezoelectric proprieties, wide signal bandwidth response, high pressure-voltage linearity, and high signal-to-noise ratio Lu et al. (2019), and it has been used as a promising candidate for the development of force sensors in miniaturized medical devices (Gil et al., 2020).

Emerging bio-inspired materials, for example, ionic polymer metal composite (IPMC) and electroactive polymer artificial muscle (EPAM) (Shi and Yeatman, 2021), offer great potential to produce the propulsion or bending forces required in many applications. The bending force of IPMC is generated by the effective redistribution of hydrated ions and water, which is best known as ionic-induced hydraulic actuation (Chattaraj et al., 2014). However, the current availability and implementation are hindered by the requirement for high-voltage power supplies.

Negative photoresist, such as SU-8, is an epoxy-based photo-patternable polymer with large elastic modulus, high glass transition temperature, and presents good biocompatibility Gyak et al. (2019). These properties make SU-8 a great choice for various *in vivo* applications, although the long-term stability in cell culture media and biocompatibility is under debate. Chronis and Wood have both developed SU-8-based micro-grippers (Wood et al., 2014), which show their promising potential in the manipulation of cells and other biological entities. Stimuli-responsive materials can naturally provide self-assembling and self-folding capabilities to micro-robots, which eliminates the need for using bulky batteries (Ongaro et al., 2016b). In this regard, improved materials property is vital to micro-robotics systems. The other intelligent materials will be introduced in Section 3.5.

### 3.2 Fabrication and assembly techniques

For micro-robotics, all on-board components such as actuators, sensors, energy, or power units must be miniaturized to micro-scale. This requires dedicated micro-fabrication techniques.

The methods for the fabrication of microelectromechanical systems (MEMS) have enabled the rapid development of micro-robotics. MEMS allows the precise production of micro-scale features made from different materials while showing both compactness and compatibility with current industrial technologies. Surface and bulk micro-machining are among the most commonly used techniques for the fabrication of micro-robotics. For example, Volland et al. reported the micro-actuators and micro-grippers fabricated from SOI (silicon-on-insulator) wafers by using surface and bulk micro-machining (Volland et al., 2002). The advantages of this method mainly include 1) the high stability against disturbing forces and large thickness and 2) fewer fabrication steps. Lithography presents another major method that has been widely used for the fabrication of micro-robotics. As a milestone work, Kim et al. demonstrated the fabrication of three-dimensional (3D) porous structures using a 3D laser lithography system (Kim et al., 2013). After the 3D photolithography process, the micro-structures are coated with nickel and titanium films for magnetic manipulation purposes and to enhance biocompatibility. The resulting micro-robotics have been successfully demonstrated in transporting human embryonic kidney 239 (HEK 239) cells in the fluid using external magnetic fields. In addition to these two methods, laser micro-machining (Bordatchev and Nikumb, 2003), LIGA (Lithography, Electroplating, and Molding) (Menciassi et al., 2003), template electrodeposition (Li et al., 2022), thin film deposition (Hanson et al., 2022), and electro-discharge machining (EDM) (Kim et al., 2005a) have brought many concepts of micro-robots into reality.

Due to rapid technological development, 3D printing techniques have become the most promising methods for the fabrication of micro-robotics with complex shapes, helping to enhance their capabilities in conducting dexterous tasks for both *in vivo* and *in vitro* applications. 3D printing can be categorized into digital light processing (DLP) (Li and Pumera, 2021), stereolithography (SLA) (Zhan et al., 2021), fused deposition modeling (FDM) (Ben Salem et al., 2019), selective laser sintering (SLS) (Exner et al., 2008), two-photon polymerization (TPP) (Power and Yang, 2015), direct laser writing (DLW) (Tottori et al., 2012), direct metal laser sintering (DMLS) (Wong and Hernandez, 2012; Guo and Leu, 2013) and other direct writing methods (Teh, 2017). As an example, Zhang et al. developed micro-robots using the TPP technique, which can be manipulated using planar multi-spot optical tweezers. Such a versatile platform can be used for the manipulation of living cells or organisms on the microscopic scale without damage to them from direct laser illuminating. 3D printing is excellent for prototyping, but less well suited to high volume, low cost production. Therefore, advanced micro-fabrication techniques for mass production are worth developing.

### 3.3 Actuation of micro-robotics

Actuation is essential for micro-robots in terms of both locomotion and manipulation. Effective actuation can be achieved through the usage of advanced fabrication and the selection of proper materials. Many different types of material have been used for actuation in micro-robotics, including electrostatic (Xu, 2015), piezoelectric (Nah and Zhong, 2007), pneumatic (Alogla et al., 2015), or thermal actuators (Qu et al., 2015), and hybrid actuation systems (Chen et al., 2010b). Hybrid actuation represents the integration of different actuation modes, which combines the relative advantages of different actuation schemes for manipulating micro-robots.

Advances in miniaturized power and energy systems may also bring technological breakthroughs to the actuation of micro-robots. For example, rechargeable thin-film arbitrarily shaped batteries fabricated through semiconductor technologies can be used for micro-robots, since batteries for large-scale robots are not applicable to micro-scale robots. AbuZaiter et al. developed a novel structure of a SMA based micromanipulator for gripping purposes (Abuzaiter et al., 2016). The integration of multiple SMA bimorph actuators has been employed to manipulate objects with three degrees of freedom (DoFs). This novel design includes two links and a micro-gripper at the end of the second joint.

In addition, innovative actuation strategies have also been developed in parallel for actuating micro-robotics. For example, freeze tweezers, which are based on the Joule-Thompson throttling effect, can be used to operate micro-objects (Walle et al., 2007). Liu et al. reported a freeze tweezer using the nucleotide ice ball to manipulate micro-objects in an aqueous solution. By tuning the freezing parameters, the ice balls formed between the tweezer tip and the object can be used as contacts to pick up objects (Liu et al., 2003), where the release process during the micro-manipulation is challenging due to the sticking effect.

To tackle this challenge, non-contact manipulations have been proposed and are considered as the most promising techniques (Hattori et al., 2015). As an example of chemical controlled manipulation, Randhawa’s group presented the concept of single-use, chemically triggered, and reversible micro-grippers, which can be operated by acetic acid controlled “open” and hydrogen peroxide controlled “close” options (Randhawa et al., 2008). In addition to the chemical-controlled manipulation, the manipulation can be more precisely controlled through the finely tuned field forces. For example, magnetic fields (Chung et al., 2015), acoustic waves (Kim et al., 2015), dielectrophoresis (DEP) (Grilli and Ferraro, 2008), fluid inertial forces (Grilli and Ferraro, 2008), or microbubble streaming can be used to actuate microrobots’ microbubble (Lee et al., 2013). Zhang et al. reported the artificial bacterial flagella, which consists of a helical InGaAs/GaAs or InGaAs/GaAs/Cr tail and a thin soft-magnetic head. Such a micro-robot can swim and be manipulated in the controllable magnetic fields (Zhang et al., 2009), which represents the first generation of artificial swimmers using helical propulsion. The controlled locomotion of untethered micro-robots is attractive both for fundamental research and for biomedical applications (Sitti et al., 2015). The future development trend is to achieve fully wireless-driven manipulators for medical and biological applications under 3D control with a full six DoFs.

### 3.4 Sensing capability in micro-robotics

To avoid damage to fragile biological objects during micro-manipulation, an integrated sensing system is also required. Many micro-sensors have been designed and integrated as a part of the manipulation system. For example, Petrovic et al. developed an optical sensing system to estimate the force feedback from each mechanical micro steel arm in the micro-grippers (Petrovic et al., 2002). The integrated optical sensor includes a LED for light emission, a photosensitive element as the light receptor, and the corresponding electronics for signal processing. After calibration, the relation between the output voltage from the optical sensor and the force exerted on the tooltip of the micro-gripper can be precisely correlated. Zhu et al. reported an on-chip electrothermal sensor for high precision measurement with resolution at nanometer scale (Zhu et al., 2011).

Capacitive force sensors have been used to measure the adhesive forces between the micro-gripper fingers and the applied force (Beyeler et al., 2007). Unlike the high-resolution optical force sensor, this capacitive sensor does not require complicated setup to produce a sensitivity of 4.41 mV/*μ*N with resolution of 70 nN. Beyeler et al. used this micro-gripper to successfully demonstrate the capacity to manipulate HeLa cells. On the other hand, piezo-resistive sensors are also capable of measuring the gripper deflection to estimate the applied forces. Molhave’s group demonstrated an electrothermally actuated micro-gripper with a capability of providing a force-feedback signal using a piezo-resistive sensor (Molhave and Hansen, 2005). This sensor is based on a simple structure consisting of three parallel beams connected by an end bar, which shows a high resolution of 0.1 mV/*μ*N in the precise gripping of nanoscale objects. Other types of micro-sensors have been comprehensively discussed with performance metrics comparison amongst them (Wei and Xu, 2015). However, most of the micro-sensors are integrated with tethered micro-robots. As for micro-robots navigated inside human bodies, more advanced sensing systems are worth developing.

### 3.5 Intelligence for micro-robots

#### 3.5.1 Physical intelligence

Sensing capabilities are significant for micro-robots to interact with the environment intelligently (Soto et al., 2021). Using smart materials with instinctive properties (Martella et al., 2017), embedded intelligence (also known as physical intelligence) can be realized by integrating sensing and actuation capabilities in micro-robots and enabling them to respond to local environment accordingly. These abilities can be achieved by using smart materials (Khoo et al., 2015), including hydrogels, shape memory polymers (SMPs), liquid crystal elastomers (LCEs), etc.

Hydrogels can enable micro-grippers with biocompatibility functionality for use in many biomedical applications (Kuo et al., 2014). These materials can deform significantly in response to a variety of stimuli, such as pH, temperature, light, or chemical reactions, thus generating enough energy and force to perform manipulation tasks (Power et al., 2017). A hydrogel-based intravascular micro-gripper manipulated using magnetic fields has been proposed in (Kuo et al., 2014). The material is biocompatible, while the actuation of the gripper was implemented by controlling the current passing through the coils to alter the magnetic field. This mechanism can be realized by controlling the exposure dose on the hydrogel composite during fabrication process. Hydrogels can also be used in combination with stiff polymers to achieve self-folding characteristics. Operation and functionality of polymeric micro-grippers for soft robotics in surgical applications have been described in previous reports (Breger et al., 2016).

To govern the locomotion of soft robots, autonomous regulation of fluid flow can be implemented with micro-fluidic logical operators (Wehner et al., 2016). Magnetic micro-robots have been developed to change their shape to avoid being washed away by a flow through the sensing of flow rates (Wang et al., 2021). A soft micro-robot has integrated engineered bacteria for chemical sensing, which enables autonomous gripping motions based on the detecting chemicals (Justus et al., 2019). A 2D carbon nitride-based Janus micro-robot has demonstrated its ability to propel itself in aqueous media by making use of the presence of light (Sridhar et al., 2020). These micro-robots are considered to have embedded intelligence.

More recently, the advent of 4D printing technologies can accelerate the development of micro-robots with physical intelligence. Instead of fabricating static solid micro-robots, 4D printing can enable micro-robots to change their structural shape and/or properties after being printed, which can be activated by external energy or environmental stimuli. To this end, micro-robots have adaptive, shape-shifting abilities for complex biomedical applications. Recent advances in 4D printing technologies for micro-robotics can be found in (Adam et al., 2021).

#### 3.5.2 Artificial Intelligence

Machine learning methods have demonstrated great performance in the design, modeling, perception and manipulation of micro-robots. For example, machine learning can be used to optimize the fabrication process of micro-robots (Jin et al., 2019), and to track and estimate the pose of micro-robots (Zhang, 2021), which paves a way for autonomous control of micro-robots. For example, Convolutional Neural Networks (CNNs) have been used for depth and 3D orientation estimation of micro-robots observed *via* microscope (Grammatikopoulou and Yang, 2019). A deep residual neural network and Gaussian Process Regression (GPR) hybrid model has been proposed, which can estimate the six DoFs pose of micro-robots (Zhang et al., 2020a). However, most of the deep learning-based methods require the collection of a large database. Therefore, simulation data has been combined with experimental data to implement a sim-to-real learning-to-match approach for pose estimation of micro-robots (Zhang et al.,2022).

For real-world applications, micro-robots need to navigate in non-Newtonian fluidic environments, where the dynamic properties are difficult to model. Due to the absence of onboard sensors, practical disturbances and uncertainties may bring extra challenges to precise control of micro-robots. In this case, reinforcement learning can be used for micro-robots to learn the policy through interaction with the environment, through which the micro-robots can accumulate experience of navigating in complex fluid flows and adapt to different scenarios (Colabrese et al., 2017).

Unavoidable Brownian motion at microscopic scales can randomly perturb the position and propulsion direction of micro-robots. To tackle this issue, a Q-learning method has been used for light-driven micro-robots to form adaptive behavior in a noisy environment during targeted navigation task (Muiños-Landin et al., 2021). Results indicated that with the reinforcement learning approach, the self-thermophoretic micro-robot can generate reasonable actions to solve the standard navigation problem in a real-world environment. Q-learning has drawbacks since the system states are discrete. Deep Q-learning approaches have been developed to control the motion of a colloidal micro-robot towards a target, while avoiding obstacles in the environment (Yang et al., 2020a). Furthermore, an actor-critic network has been employed for a reinforcement learning-based controller for various types of micro-robots, which enable them to fulfill the targeted locomotion tasks (Yang et al., 2020b).

## 4 Development trend of medical micro-robots

**FIGURE 3:**
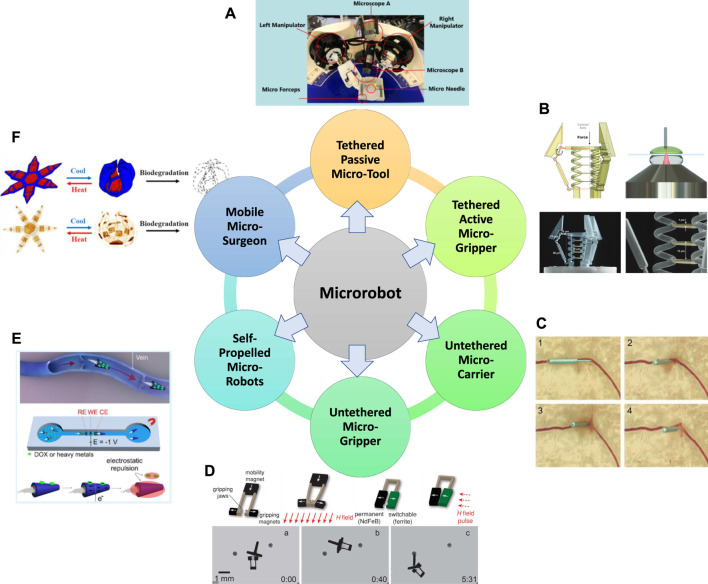
Overview of the six major categories of micro-robot built to date. **(A)** Tethered passive micro-tools (Zhang et al., 2020b; Zhang et al., 2020c), **(B)** Tethered active micro-grippers (Power et al., 2018), **(C)** Untethered micro-carriers (Chatzipirpiridis et al., 2015a), **(D)** Untethered micro-grippers (Diller and Sitti, 2014), **(E)** Self-propelled micro-robots (Beladi-Mousavi et al., 2019), **(F)** Mobile micro-surgeons (Kobayashi et al., 2018). Panel **(A)** reproduced from (Zhang et al., 2020b) under the terms of the CC-BY license. Copyright 2020, The Authors, published by Springer Nature. Panel **(B)** reproduced from (Power et al., 2018) under the terms of the CC-BY license. Copyright 2018, The Authors, published by Wiley. Panel **(C)** reproduced from (Chatzipirpiridis et al., 2015a) with permission. Copyright 2015, Wiley. Panel **(D)** reproduced from (Diller and Sitti, 2014) with permission. Copyright 2014, Wiley. Panel **(E)** reproduced from (Beladi-Mousavi et al., 2019) with permission. Copyright 2019, American Chemical Society. Panel **(F)** reproduced from (Kobayashi et al., 2018) with permission. Copyright 2019, American Chemical Society.

In the past few decades, there are emerging micro-robotic platforms developed with different characteristics. Based on the capability for locomotion, manipulation, on-board actuation, and self-propulsion, the existing micro-robotic systems are classified into six categories in this paper, which will be detailed in this section. The classification of micro-robots reveals the development of micro-robotics from tethered to untethered systems, from simple assistive tools to grippers with actuators and sensors, and from passive operation to active operation, where Figure 3 demonstrates the typical examples. Functionalities of the six major categories of micro-robots are summarized in Table 1.

**TABLE 1:** Functionalities of the six major categories of micro-robots.

Categories	Manipulation	Locomotion	On-board actuation	Self-propulsion
Tethered passive micro-tools	●	—	—	—
Tethered active micro-grippers	●	—	●	—
Untethered micro-carriers	●	●	—	—
Untethered micro-grippers	●	●	●	—
Self-propelled micro-robots	—	●	—	●
Mobile micro-surgeons	●	●	●	●

Locomotion (or global actuation) represents the ability of a micro-robot to move from one location to another for task execution, while manipulation represents the ability of the micro-robot to interact with other objects. On-board actuation means that the micro-robot has assembled actuators that can generate relative motions to grasp or release objects. Self-propulsion means the micro-robots have on-board energy supply, while the other micro-robots are actuated by remote physical fields, such as magnetic, acoustic, optical, or electrical fields to ensure flexible locomotion.

### 4.1 Tethered passive micro-tools

Tethered passive micro-tools are normally linked to a large-scale robotic platform for global position control or an energy delivery device. Tethered passive micro-tools such as micro-probes, micro-tweezers (Grier, 2003; Ghadiri et al., 2012), micro-tips (Xie and Régnier, 2011), and micro-pipettes (Lu et al., 2010; Bergeles and Yang, 2014) are capable of handling tiny objects (Wason et al., 2012), but rely on bulky and expensive setups (Chronis and Lee, 2005) to realize their functions without on-board actuators.

Tethered micro-robotic systems are effective tools for micro-assembly tasks, since they can ensure high precision and flexibility compared to manual operation methods. For example, a system with two tethered micro-tools has been developed to automatically grasp micro-modules to achieve bottom-up assembly of cell-laden hollow micro-tubes, which can also be used to construct other biomimetic structures with micro-structural features (Wang et al., 2016). A seven DoFs haptic interface was used to control the motions of the tethered micro-tools remotely, which can ensure safety during the micro-manipulation and enhance efficiency by providing intuitive haptic feedback to the operator (Feng et al., 2022).

More recently, a novel compliant tethered passive micro-tool has been developed for closed-loop cooperative manipulation, which was fabricated using the TPP micro-fabrication technique. It has the advantages of high flexibility, robustness, and increased workspace for both 2D and 3D object manipulation tasks. It can be used for compliant object manipulation without causing damage to delicate micro-objects (Power and Yang, 2015).

### 4.2 Tethered active micro-grippers

Similar to tethered passive micro-tools, tethered active micro-grippers are attached to a large-scale robotic system, but have enhanced flexibility for target object manipulation. They are typically constructed through two controllable cantilevers with on-board actuators, which can be used to grasp, clamp, or squeeze micro-objects.

As for actuation methods applied to micro-grippers, there are many different types, including electrostatic (Xu, 2015), piezoelectric (Nah and Zhong, 2007), pneumatic (Alogla et al., 2015), thermal actuators (Qu et al., 2015), SMA actuators (Munasinghe et al., 2016), or hybrid actuation systems (Chen et al., 2010b). Freeze tweezers based on the phase-changing principle can also be used (Walle et al., 2010) to operate micro-objects.

Many tethered active micro-grippers have integrated actuation and sensing capabilities (Kim et al., 2008). A novel piezoelectric actuated compliant tethered micro-gripper has been developed, which featured precise position tracking, high-speed control, and robust force regulation. This tethered active micro-gripper can be used for fast, accurate, and reliable micro-manipulation (Wang et al., 2016). Other tethered micro-grippers with integrated actuation and sensing capabilities have been reported. These include an electrostatic micro-gripper with capacitive sensor (Beyeler et al., 2006), a MEMS-based micro-gripper with electro-thermal actuators and integrated electro-thermal force sensor (Piriyanont et al., 2013), a micro-gripper with integrated electromagnetic actuator and piezoelectric force sensor (Kim et al., 2005b). The differences among those actuation and sensing strategies in terms of advantages, disadvantages, and working principles are described in another review paper with more details (Yang and Xu, 2017).

More recently, optical and multi-material fibres have provided new solutions for developing integrated devices, as they can deliver a wide range of energy levels to body tissues and have attractive features such as flexibility and cost-effectiveness in applications for microsurgery. A monolithic force-sensitive 3D micro-gripper has been designed and fabricated on the tip of a fibre using the TPP method, the dimensions of which are less than 100 *μ*m in all three axes. It is a compliant gripper with an integrated force sensor and has a high potential for the interrogation of biological microstructures such as alveoli, villi, or even individual cells (Power et al., 2018). It also has the potential for deployment in minimally invasive microsurgical tasks or biopsy. A fluid-pressure actuated micro-gripper has been fabricated using an *in situ* DLW (isDLW) technique, using IP-L 780 material (Alsharhan et al., 2021). When pressure was not present, the micro-grippers remained open. The inward movement of micro-grippers was triggered by the input pressure. With different pressure inputs, the flow modes inside these microfluidic devices could be altered (Alsharhan et al., 2021).

Tethered passive micro-tools and tethered active micro-grippers are usually tethered by wires or tubes, which restrict their maneuverability when they are required to access remote environments such as inside the human body, or enclosed environments such as lab-on-a-chip devices. Therefore, untethered micro-robots are worth developing, which can make good use of wireless power transfer, or on-board power, to enable flexible locomotion or manipulation in human anatomy or confined environments.

### 4.3 Untethered micro-carriers

Untethered micro-carriers can also be considered as untethered passive micro-tool, microscopic artificial swimmers (Dreyfus et al., 2005), untethered mobile micro-robots, or untethered micro-swimmers (Kim et al., 2015), whose locomotion capabilities are enabled by magnetic, acoustic, optical, or other remote physical fields for non-contact actuation (Li et al., 2017).

Light-controlled thermoplasmonic untethered micro-carriers have been developed for indirect manipulation of colloidal particles (Engay et al., 2018). Synthetic micro carriers powered by ultrasound have been reviewed in (Rao et al., 2015), while bioinspired magnetic micro-carriers have been introduced in (Bente et al., 2018). Acoustically and magnetically controlled untethered micro-carriers, consisting of micro-capsules with air bubbles trapped inside, have been fabricated by the TPP technique and coated with nickel and gold (Ren et al., 2019). They can be used for indirect single-particle manipulation, such as cell manipulation.

Untethered micro-carriers are promising for clinical translation. For example, an implantable magnetic tubular micro-robot can serve as a targeted drug delivery system in the posterior segment of the eye (Chatzipirpiridis et al., 2015b). Its mobility and controllability have been verified through experiments inside the eyes of a living rabbit (Chatzipirpiridis et al., 2015b). For targeted drug delivery, biodegradable metal-organic framework (MOF) based robots (MOFBOTs) have been developed through the combination of magnetic micro-robots and MOFs, while selective degradation in cell cultures can be achieved. To this end, flexible motility and high drug loading efficiency can be ensured for clinical applications (Terzopoulou et al., 2020).

### 4.4 Untethered micro-grippers

Untethered micro-grippers should be built upon untethered micro-carriers to handle challenging tasks where manipulation is required. Different from untethered micro-carriers, untethered micro-grippers have on-broad actuators to provide gripping motions. They can perform more complex tasks with a high dexterity by making good use of integrated sensing and control systems. Untethered micro-grippers can capture and retrieve tissue or cells from hard-to-reach places that are previously infeasible. This represents an important step toward non-invasive microsurgery (Vikram Singh and Sitti, 2016).

Untethered micro-grippers require external devices for wireless power transmission to realize non-contact manipulation (Zhang and Diller, 2016; Zhang et al., 2017). An electrostatically driven untethered micro-gripper for blood vessel manipulation has been developed, which can be used for the automated study of blood vessel wall contraction forces (Wierzbicki et al., 2006). Magnetically-actuated micro-grippers have been designed for precise manipulation, which can pick and place individual microgels to target areas for assembly tasks in aquatic environments (Chung et al., 2015). These untethered micro-grippers are promising for biomedical applications such as tissue reconstruction.

Untethered micro-grippers can make diagnostic and therapeutic procedures less invasive than ever before (Nelson et al., 2010) since they can navigate through natural pathways in the human body to conduct wireless intervention (Sitti et al., 2015). For example, a hydrogel-based untethered micro-gripper actuated *via* a magnetic field has been developed, which is targeted for intravascular applications (Kuo et al., 2014). A thermally-driven untethered micro-gripper has also been developed (Gultepe et al., 2013a), which can navigate through narrow conduits in the human body and be used for localized biopsies. These have a high potential to be applied for complex tasks that require the cooperation of multiple micro-grippers (Zhang et al., 2017).

### 4.5 Self-propelled micro-robots

When it comes to power transmission for self-propelled micro-robots, one of the most favorable strategies would be to design micro-robots to harvest energy from the environment. For example, they can be powered by the chemical energy inside the cell or in the microfluidic environment. Other diverse environmental stimuli such as magnetotaxis, galvanotaxis, phototaxis, thermotaxis, and aerotaxis can be used to control the robot.

Most of the self-propulsion capabilities of micro-robots are enabled by chemical reactions. Micro-robots fabricated by catalytic materials can move quickly in an acidic environment, since the generation of continuous streams of bubbles can be used as thrust to propel and steer the micro-robot. These micro-robots can then be decomposed after task execution, and the process of retrieval is no longer necessary.

Zinc-fueled micro-robots have been fabricated, which can navigate inside the stomach of a mouse. The gastric acid reacted with a section of the micro-robot to generate non-toxic oxygen bubbles that provide propulsion (Gao et al., 2015). With the support of stimuli-responsive materials, micro-robots can generate autonomous responses in specific environments (Peraza-Hernandez et al., 2014) through self-folding (Ionov, 2013). A stimuli-responsive hybrid micro-robot fabricated by TPP has been developed (Power et al., 2017), which uses two independent actuation schemes (pH- and photo-responsive actuation systems) to control the motions of the micro-robots. It can be employed for the localization of specific cell types and target drug delivery. Related experiments have demonstrated autonomous navigation towards the target with a low-pH source in a low-flow *in vitro* microfluidic environment.

### 4.6 Mobile micro-surgeon

An untethered mobile micro-surgeon is known as the combination of a mobile micro-platform and a micro-manipulator (including end-effectors such as micro-grippers), which means that it has both manipulation and locomotion capabilities. Moreover, mobile micro-surgeons have on-board actuators, and can be self-contained and self-propelled. To this end, untethered mobile micro-surgeon can handle non-trivial tasks, and are promising to detect and combat pathologies such as cancer.

A self-folding untethered micro-robot has been developed for cell delivery (Fusco et al., 2014), which shows potential for biomedical applications such as microsurgery, on-demand drug and cell delivery. A soft thermally responsive magnetic micro-robot has been introduced in (Ongaro et al., 2016a), which can conduct pick-and-place tasks by altering both the magnetic field gradient and the temperature in the workspace of micro-robots. Biohybrid robots, developed using biomaterials, have a high potential to become mobile micro-surgeons, since they utilize power from living organisms or cells for locomotion and manipulation, while on-board actuation can be achieved due to biological activity. They have high power density and controllability (Lin et al., 2021).

Red blood cells (RBCs) have been attached to bioengineered motile bacteria to construct biohybrid robots, where RBCs were used as cargo carriers while propulsion was enabled by bacteria, with external magnetic guidance. These biohybrid micro-robots can be known as promising multimodal targeted cargo delivery systems, since they have remarkable advantages, including biocompatibility, deformability, stability. Bacteria-driven biohybrid robots have demonstrated their capabilities for active drug delivery to tumors or other target areas. More recently, Volbot (Wang et al., 2022), a type of biohybrid volvox-based micro-robot, has been developed with self-propelled capability. The biohybrid Volbot can be known as an all-in-one multifunctional micro-robot, since it has autonomous phototaxis navigation and multimode imaging capabilities. It demonstrates a high potential for applications in precision tumor therapy. More details about biohybrid micro-robots, their applications as well as the future outlook can be found in (Lin et al., 2021).

## 5 Applications of medical micro-robots

**FIGURE 4:**
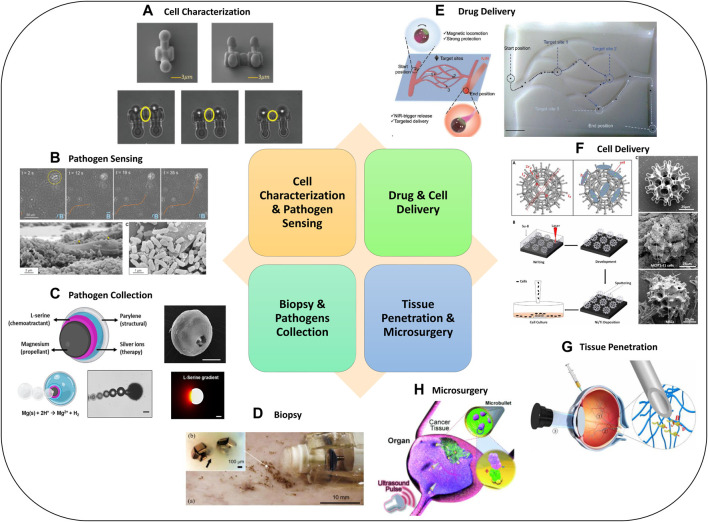
Advanced Micro-robotics for biomedical applications. Examples for **(A)** cell characterization (Zhang et al., 2020d), **(B)** pathogen sensing (Stanton et al., 2017), **(C)** pathogen collection (Soto et al., 2020), **(D)** biopsy (Yim et al., 2014), **(E)** drug delivery (Song et al., 2022), **(F)** cell delivery (Li et al., 2018), **(G)** tissue penetration (Wu et al., 2018), **(H)** microsurgery (Kagan et al., 2012). Panel **(A)** reproduced from (Zhang et al., 2020d) under the terms of the CC-BY license. Copyright 2020, The Authors, published by Wiley. Panel **(B)** reproduced from (Stanton et al., 2017) with permission. Copyright 2017, American Chemical Society. Panel **(C)** reproduced from (Soto et al., 2020) with permission. Copyright 2020, Wiley. Panel **(D)** reproduced from (Yim et al., 2014) with permission. Copyright 2014, IEEE. Panel **(E)** reproduced from (Song et al., 2022) under the terms of the CC-BY license. Copyright 2022, The Authors, published by Wiley. Panel **(F)** reproduced from (Li et al., 2018) with permission. Copyright 2018, published by The American Association for the Advancement of Science. Panel **(G)** reproduced from (Wu et al., 2018) under the terms of the CC-BY license. Copyright 2018, The Authors, published by The American Association for the Advancement of Science. Panel **(H)** reproduced from (Kagan et al., 2012) with permission. Copyright 2012, Wiley.

Micro-robots can be used for medical diagnosis. For example, they can isolate pathogens or measure physical proprieties of tissue in real-time. They can also navigate to target regions and deliver a precise dosage of drug to retain their therapeutic efficacy while reducing side effects. Moreover, they can reach hard-to-reach areas that are not accessible by catheters or invasive surgical tools, which enables biopsy and microsurgery and reduces the invasiveness of surgical procedures. In this section, we introduce the typical applications of medical micro-robots, while typical examples were shown in Figure 4.

### 5.1 Cell characterization and pathogen sensing

Single-cell analysis and characterization are important for new drug discovery and other relevant biomedical applications, which can be known as the killer applications for micro-robots. These applications are known as *in vitro* applications, since operations are conducted in laboratories outside the human body (Ahmad et al., 2021) and do not involve most of the clinical challenges as mentioned in Section 2.3. Kawahara et al. developed the quantitative evaluation of the stimulation of Pleurosira laevis and determined the relationship between the applied force and the response of a single cell (Kawahara et al., 2013). Predefined applications such as tumor-cell detection and capture tasks can be completed by untethered micro-robots that have been released into the biomedical sample (Hajba and Guttman, 2014).

Micro-robots can be used as effective tools to identify mechanical characteristics of cells, since micro-robots have advantages such as high throughput and repeatability compared to manual approaches. An untethered micro-robot equipped with a micro force sensor has been developed and used for cell positioning and sensing after force sensing calibration. The force-controlled manipulation capability of the micro-robot can ensure that the cells are positioned properly without damage or rupture (Jing et al., 2018). Magnetically driven on-chip micro-robots have been developed to investigate the response of aquatic microorganisms (*Pleurosira laevis*) quantitatively when providing mechanical stimulation, through which the relationship between the applied force and the response of a single cell can be analyzed (Kawahara et al., 2013).

To realize early interventional therapy, pathogens should be analyzed with rapid evaluation. Micro-robots can be effective tools to reach this target (Draz et al., 2018). Fluorescent magnetic spore-based micro-robots (FMSMs), synthesized by the direct deposition of magnetic nanoparticles, have been used as a sensing platform for toxins secreted by *Clostridium difficile* (*C. diff*). The sensing probes were encapsulated on the porous natural spores of micro-robots (Zhang et al., 2019). A biohybrid magnetic micro-robot was developed as a drug-loaded silica microtube that could be steered toward the target biofilm colony and kill *E. Coli*. This micro-robot consists of a magnetically guided bacteria integrated inside a drug-loaded silica microtube (Stanton et al., 2017).

### 5.2 Biopsy and pathogens collection

Tissue biopsy coupled with the histopathologic examination is the gold standard for non-invasive diagnosis of diseases such as cancer or inflammatory diseases (Gultepe et al., 2013b), where untethered endoscopic micro-grippers can be used to retrieve tissue samples for diagnostics.

Micro-robots have been developed as effective tools for both *in vitro* and *in vivo* biopsy. For example, a set of responsive micro-grippers have been fabricated for the removal of cells from tissue embedded at the end of a capillary (Leong et al., 2009). These micro-grippers were mass-producible, while the actuation can be remotely triggered by chemicals and temperature to generate motions for *in vitro* biopsy, such as removing cells from tissue embedded at the end of a capillary.

More recently, untethered micro-robots are promising to conduct *in vivo* biopsy. In (Yim et al., 2014), a magnetically actuated untethered soft capsule endoscope has been developed, which can carry and release thermo-sensitive untethered micro-grippers (*μ*-grippers). These untethered micro-robots offer multi-functional strategies for gastrointestinal capsule biopsy. A stimuli-responsive soft gripper for chemomechanical controlled release was developed by Malachowski et al., which can elute chemotherapeutic drug doxorubicin while grabbing a clump of breast cancer cells (Malachowski et al., 2014).

Micro-robots can also be used for pathogen collection. A micro-robot consisting of an onion inspired multilayer structure has been developed to collect motile pathogens (Soto et al., 2020). A hollow structure serves as a trap to store the target samples, while the inner layer structure releases a chemoattractant to attract and capture motile microorganisms and store them in the hollow structure (Soto et al., 2020). However, the challenges of preserving the specimens and protecting them from contamination remain.

### 5.3 Drug and cell delivery

Due to their small size and flexibility, micro-robots are playing an increasingly important role for targeted drug delivery and cell delivery. Several typical examples of stimuli-responsive soft untethered grippers for drug delivery have been reviewed in (Ghosh et al., 2017). Dual-action micro-daggers were developed in (Srivastava et al., 2016), which can create a cellular incision followed by the drug release feature for localized drug administration. An electromagnetically actuated micro-robot has been developed for targeted drug delivery (Jeong et al., 2020), while the drug control process, including carrying, releasing, and penetrating, can be achieved by acoustically oscillating two bubbles with different natural frequency.

The *in vivo* study of chemically powered micro-motors has been conducted by Gao et al. (Gao et al., 2015). The body of the micro-motors can dissolve in the gastric acid gradually. The payloads carried by them were released autonomously without leaving toxicity behind. *In vivo* imaging and actuation of a swarm of helical micro-swimmers under rotating magnetic fields in deep tissue was developed and introduced by Servant et al. (Servant et al., 2015). The motions of the robots were tracked in real-time using fluorescence imaging. This demonstrates the possibility of optimal drug delivery to a targeted site guided by the external magnetic field (Servant et al., 2015).

As for cell delivery, magnetic field–driven micro-robots with a burr-like porous spherical structure have been introduced in (Li et al., 2018), which can serve as a promising technique for targeted therapy and tissue regeneration. The micro-robots can deliver hippocampal neural stem cells with proliferation and differentiation capabilities. To this end, *in vivo* cell releasing tests were conducted on mice to demonstrate its potential for cell-based therapy (Li et al., 2018).

A self-folding micro-robot, which can be used for cell delivery, has been described in (Fusco et al., 2014). Cells carried by micro-robots have been shown to reach the desired site during *in vitro* experiments and passing through the blood vessel-like micro-channel to arrive at the delivery site. By precisely delivering the differentiated stem cells at the pathological sites (Fox et al., 2014) or culturing human cells in 3D inside the micro-robot and using them as bio-scaffold for tissue regeneration, many diseases could be treated.

### 5.4 Tissue penetration and microsurgery

To fulfill the target of *in vivo* micro-surgery, untethered soft micro-grippers have significant advantages, due to the compliant motions and the capability of handling delicate objects (Rus and Tolley, 2015). They can carry cargoes and navigate in complex anatomy with varying geometries and dimensions, while being adaptive in environments where rapidly changing physiological conditions, soft tissues, and unanticipated biological events are involved.

Micro-robots can be actuated by ultrasonic power in bullet-like manner through acoustic droplet vaporization-ignition mechanisms. For example, micro-robots consisting of a hollow tube filled with perfluorocarbon emulsions can vaporize and rapidly change their state from liquid to gas to generate thrust (Kagan et al., 2012). The remarkable high speed can benefit applications, such as ablation, deep tissue penetration, etc., (Kagan et al., 2012).

An untethered magnetic micro-robot has been developed and used as an implantable tubular micro-tool for wireless ophthalmologic application (Chatzipirpiridis et al., 2015a). The micro-robot can be injected into the central vitreous humor of a living rabbit eye using a micro-needle and can be observed by an ophthalmoscope and integrated camera. More recently, micro-robots in ocular surgery have demonstrated potential applicability (Wu et al., 2018). A multi-functional biohybrid magnetite micro-robot has been developed for image-guided therapy, which has addressed the critical technical issues such as motion tracking, biocompatibility, biodegradation (Li et al., 2020), and diagnostic/therapeutic effects with preclinical *in vivo* experiments on subcutaneous tissue and the intraperitoneal cavity of nude mice.

## 6 Future outlook

Micro-robots can be developed into powerful tools for a variety of biomedical applications. However, their development and use is still limited as their clinical feasibility has not yet been widely verified. Furthermore, being able to monitor, manufacture, and robustly actuate them are major challenges.

To realize the full potential of micro-robots, it will be necessary to expand their operational abilities to support more complex tasks. This will require smart materials with versatile properties, delivery of wireless energy through untethered power systems, and improved reliability. In the meantime, new actuation, sensing, control, and manufacturing methods should be studied for future development. More advanced materials need to be developed to meet the requirement for future micro-robotics, such as propulsion, biocompatibility, intelligence, and cooperation. These materials may have self-adaptability, self-sensing, or memory capabilities, which potentially offer micro-scale programmability by directly integrating intelligence into the actuators, valves, and other cooperative or biocompatible structures. Therefore, it has become one of the most important research areas to study the biological and physicochemical properties of smart materials to incorporate the required functionalities for intelligent programmable micro-robotics with distinct capabilities.

Another avenue that is starting to see promise is the engineering of biology itself to address some of the challenges facing micro-robotics research. In particular, the re-purposing of living cells as controllable or programmable entities (Greco et al., 2019; Marucci et al., 2020) can act as a basis for “living micro-robots”. A major advantage of this approach is access to the vast innate capacity of cells to sense and actuate at the micro-scale, as well as self-propagate to huge numbers at little cost. While the reliable reprogramming of cells is still lacking (Brophy and Voigt, 2014), some progress has been made through the use of external magnetic and light signals to control the motion of cellular collectives for medical interventions (Schuerle et al., 2019; Rubio Denniss et al., 2022a; Rubio Denniss et al., 2022b; Wijewardhane et al., 2022), as well as innate capability of cells to target cancerous environments for drug delivery (Din et al., 2016; Chowdhury et al., 2019). Micro-robots could further be interfaced with living cells by using cybergenetics platforms, where cells can be controlled in real-time using microscopy platforms, light or chemical stimuli (Pedone et al., 2021; Khammash, 2022). These advances are promising, however, the development of living micro-robots also raises new concerns regarding the safety of using self-replicating systems that are difficult to contain. The possibility for unwanted immune responses in medical applications, and difficulties around precise control and scale-up issues, all acting as barriers to their adoption (Gorochowski et al., 2020; Gorochowski et al., 2021). Even so, this is an interesting future direction for the field, and it may be that a combination of living and non-living components may ultimately offer the ideal blend of functionalities for the effective construction of micro-robots in the near future.

As we master the design of individual micro-robots, it will be important to understand how large numbers of them can work together towards a common goal, building on principles of swarm intelligence (Hauert and Bhatia, 2014). Most biomedical applications require treating large numbers of cells, and so need similar numbers of micro-robots to have an impact. Moreover, as our ability to simulate these systems improves, we are increasingly able to consider designing digital twins of the biomedical conditions we’d like to treat to use as a way to rapidly innovate and personalize the design of micro-robots.

## 7 Conclusion

Recent advances in intelligent medical micro-robots have shown promise for future healthcare systems. The past 3 decades have witnessed important advances in micro-robotics. In this article, the fundamental, practical and clinical challenges for micro-robotics research are identified. Different types of micro-robotics systems are defined, while the assembly of state-of-the-art technologies to support further micro-robotics research is introduced. We outline the key applications for micro-robotics with the main focus on early diagnosis and therapeutic interventions, and illustrate the general trend for the development of micro-robotics.

Micro-robots have opened new avenues in biomedical applications. We highlight new research opportunities that require joint efforts from researchers in different disciplines. Though grand challenges remain, we envision that the existing challenges can be gradually solved, and micro-robots can bring undeniable social impacts with feasible clinical translation. Micro-robots have the potential to advance early diagnosis and treatment of patients. However, regulatory and market challenges limit the widespread use of micro-robots in clinical settings. In order to achieve lab-to-market transition, unmet needs that can validate the market for medical micro-robots should be identified. Initial clinical translation ventures should focus on complementing existing medical devices.

## References

[B1] Abuzaiter A. Nafea M. Ali M. S. M. (2016). Development of a shape-memory-alloy micromanipulator based on integrated bimorph microactuators. Mechatronics 38, 16–28. 10.1016/j.mechatronics.2016.05.009

[B2] Adam G. Benouhiba A. Rabenorosoa K. Clévy C. Cappelleri D. J. (2021). 4d printing: Enabling technology for microrobotics applications. Adv. Intell. Syst. 3, 2000216. 10.1002/aisy.202000216

[B3] Aghakhani A. Pena-Francesch A. Bozuyuk U. Cetin H. Wrede P. Sitti M. (2022). High shear rate propulsion of acoustic microrobots in complex biological fluids. Sci. Adv. 8, eabm5126. 10.1126/sciadv.abm5126 35275716 PMC8916727

[B4] Agrahari V. Agrahari V. Chou M. L. Chew C. H. Noll J. Burnouf T. (2020). Intelligent micro-/nanorobots as drug and cell carrier devices for biomedical therapeutic advancement: Promising development opportunities and translational challenges. Biomaterials 260, 120163. 10.1016/j.biomaterials.2020.120163 32882512

[B5] Ahmad B. Gauthier M. Laurent G. J. Bolopion A. (2021). Mobile microrobots for *in vitro* biomedical applications: A survey. IEEE Trans. Robotics 38, 646–663. 10.1109/tro.2021.3085245

[B6] Alogla A. Amalou F. Balmer C. Scanlan P. Shu W. Reuben R. L. (2015). Micro-tweezers: Design, fabrication, simulation and testing of a pneumatically actuated micro-gripper for micromanipulation and microtactile sensing. Sensors Actuators A Phys. 236, 394–404. 10.1016/j.sna.2015.06.032

[B7] Alsharhan A. T. Young O. M. Xu X. Stair A. J. Sochol R. D. (2021). Integrated 3d printed microfluidic circuitry and soft microrobotic actuators via *in situ* direct laser writing. J. Micromechanics Microengineering 31, 044001. 10.1088/1361-6439/abec1c

[B8] Aziz A. Pane S. Iacovacci V. Koukourakis N. Czarske J. Menciassi A. (2020). Medical imaging of microrobots: Toward *in vivo* applications. ACS Nano 14, 10865–10893. 10.1021/acsnano.0c05530 32869971

[B9] Bark C. Binnenbose T. Vogele G. Weisener T. Widmann M. (2002). “Gripping with low viscosity fluids,” in The Eleventh International Workshop on MICRO Electro Mechanical Systems, 1998. Mems 98. Proceedings, 301–305.

[B10] Beladi-Mousavi S. M. Khezri B. Krejcova L. Heger Z. Sofer Z. Fisher A. C. (2019). Recoverable bismuth-based microrobots: Capture, transport, and on-demand release of heavy metals and an anticancer drug in confined spaces. ACS Appl. Mater. Interfaces 11, 13359–13369. 10.1021/acsami.8b19408 30925065

[B11] Ben Salem M. Hussein H. Aiche G. Haddab Y. Lutz P. Rubbert L. (2019). Characterization of bistable mechanisms for microrobotics and mesorobotics. J. Micro-Bio Robotics 15, 65–77. 10.1007/s12213-019-00113-3

[B12] Bente K. Codutti A. Bachmann F. Faivre D. (2018). Biohybrid and bioinspired magnetic microswimmers. Small 14, 1704374. 10.1002/smll.201704374 29855143

[B13] Bergeles C. Yang G. Z. (2014). From passive tool holders to microsurgeons: Safer, smaller, smarter surgical robots. IEEE Trans. Biomed. Eng. 61, 1565–1576. 10.1109/tbme.2013.2293815 24723622

[B14] Beyeler F. Bell D. J. Nelson B. J. Sun Y. Neild A. Oberti S. (2006). “Design of a micro-gripper and an ultrasonic manipulator for handling micron sized objects. Intelligent Robots and Systems,” in 2006 IEEE/RSJ International Conference on (IEEE) (IEEE), 772–777.

[B15] Beyeler F. Neild A. Oberti S. Bell D. J. Sun Y. Dual J. (2007). Monolithically fabricated microgripper with integrated force sensor for manipulating microobjects and biological cells aligned in an ultrasonic field. J. Microelectromechanical Syst. 16, 7–15. 10.1109/jmems.2006.885853

[B16] Biganzoli F. Fassi I. Pagano C. (2005). “Development of a gripping system based on capillary force,” in ISATP 2005 The 6th IEEE International Symposium on Assembly and Task Planning: From Nano to Macro Assembly and Manufacturing (IEEE), 36–40.

[B17] Bordatchev E. V. Nikumb S. (2003). “Microgripper: Design, finite element analysis and laser microfabrication. MEMS, NANO and smart systems,” in 2003. Proceedings. International Conference on (IEEE), 308–313.

[B18] Breger J. C. Yoon C. Xiao R. Kwag H. R. Wang M. O. Fisher J. P. (2016). Self-folding thermo-magnetically responsive soft microgrippers. Acs Appl. Mater Interfaces 7, 3398–3405. 10.1021/am508621s PMC4326779 25594664

[B19] Brophy J. A. N. Voigt C. A. (2014). Principles of genetic circuit design. Nat. Methods 11, 508–520. 10.1038/nmeth.2926 24781324 PMC4230274

[B20] Cabanach P. Pena-Francesch A. Sheehan D. Bozuyuk U. Yasa O. Borros S. (2020). Microrobots: Zwitterionic 3D‐printed non‐immunogenic stealth microrobots (adv. Mater. 42/2020). Adv. Mater. 32, 2070312. 10.1002/adma.202070312 PMC7610461 32864804

[B21] Ceylan H. Yasa I. C. Kilic U. Hu W. Sitti M. (2019). Translational prospects of untethered medical microrobots. Prog. Biomed. Eng. 1, 012002. 10.1088/2516-1091/ab22d5

[B22] Chang S. T. Paunov V. N. Petsev D. N. Velev O. D. (2007). Remotely powered self-propelling particles and micropumps based on miniature diodes. Nat. Mater. 6, 235–240. 10.1038/nmat1843 17293850

[B23] Chattaraj R. Bhattacharya S. Bepari B. Bhaumik S. (2014). “Design and control of two fingered compliant gripper for micro gripping,” in International Conference on Informatics, Electronics & Vision, 1–6.

[B24] Chatzipirpiridis G. Ergeneman O. Pokki J. Ullrich F. Fusco S. Ortega J. A. (2015b). Electroforming of implantable tubular magnetic microrobots for wireless ophthalmologic applications. Adv. Healthc. Mater. 4, 209–214. 10.1002/adhm.201400256 24986087

[B25] Chatzipirpiridis G. Ergeneman O. Pokki J. Ullrich F. Fusco S. Ortega J. A. (2015a). Microrobotics: Electroforming of implantable tubular magnetic microrobots for wireless ophthalmologic applications (adv. healthcare mater. 2/2015). Adv. Healthc. Mater. 4, 208–214. 10.1002/adhm.201570011 24986087

[B26] Chen T. Chen L. Sun L. Rong W. Yang Q. Micro manipulation based on adhesion control with compound vibration. Ieee/rsj International Conference on Intelligent Robots and Systems (2010a), 6137–6142.

[B27] Chen T. Sun L. Chen L. Rong W. Li X. (2010b). A hybrid-type electrostatically driven microgripper with an integrated vacuum tool. Sensors Actuators A Phys. 158, 320–327. 10.1016/j.sna.2010.01.001

[B28] Chowdhury S. Castro S. Coker C. Hinchliffe T. E. Arpaia N. Danino T. (2019). Programmable bacteria induce durable tumor regression and systemic antitumor immunity. Nat. Med. 25, 1057–1063. 10.1038/s41591-019-0498-z 31270504 PMC6688650

[B29] Chronis N. Lee L. P. (2005). Electrothermally activated su-8 microgripper for single cell manipulation in solution. J. Microelectromechanical Syst. 14, 857–863. 10.1109/jmems.2005.845445

[B30] Chung S. E. Dong X. Sitti M. (2015). Three-dimensional heterogeneous assembly of coded microgels using an untethered mobile microgripper. Lab A Chip 15, 1667–1676. 10.1039/c5lc00009b 25714053

[B31] Colabrese S. Gustavsson K. Celani A. Biferale L. (2017). Flow navigation by smart microswimmers via reinforcement learning. Phys. Rev. Lett. 118, 158004. 10.1103/physrevlett.118.158004 28452499

[B32] Dabbagh S. R. Sarabi M. R. Birtek M. T. Seyfi S. Sitti M. Tasoglu S. (2022). 3d-printed microrobots from design to translation. Nat. Commun. 13 (1), 1–24. 10.1038/s41467-022-33409-3 36198675 PMC9534872

[B33] Děkanovskỳ L. Li J. Zhou H. Sofer Z. Khezri B. (2022). Nano/microrobots line up for gastrointestinal tract diseases: Targeted delivery, therapy, and prevention. Energies 15, 426. 10.3390/en15020426

[B34] Diller E. Sitti M. (2014). Three-dimensional programmable assembly by untethered magnetic robotic micro-grippers. Adv. Funct. Mater. 24, 4397–4404. 10.1002/adfm.201400275

[B35] Din M. O. Danino T. Prindle A. Skalak M. Selimkhanov J. Allen K. (2016). Synchronized cycles of bacterial lysis for *in vivo* delivery. Nature 536, 81–85. 10.1038/nature18930 27437587 PMC5048415

[B36] Dong R. Cai Y. Yang Y. Gao W. Ren B. (2018). Photocatalytic micro/nanomotors: From construction to applications. Accounts Chem. Res. 51, 1940–1947. 10.1021/acs.accounts.8b00249 30152999

[B37] Draz M. S. Lakshminaraasimulu N. K. Krishnakumar S. Battalapalli D. Vasan A. Kanakasabapathy M. K. (2018). Motion-based immunological detection of zika virus using pt-nanomotors and a cellphone. ACS Nano 12, 5709–5718. 10.1021/acsnano.8b01515 29767504 PMC6860978

[B38] Dreyfus R. Baudry J. Roper M. L. Fermigier M. Stone H. A. Bibette J. (2005). Microscopic artificial swimmers. Nature 437, 862–865. 10.1038/nature04090 16208366

[B39] Engay E. Bunea A. I. Chouliara M. Bañas A. Glückstad J. (2018). Natural convection induced by an optically fabricated and actuated microtool with a thermoplasmonic disk. Opt. Lett. 43, 3870–3873. 10.1364/ol.43.003870 30106904

[B40] Exner H. Horn M. Streek A. Ullmann F. Hartwig L. Regenfuß P. (2008). Laser micro sintering: A new method to generate metal and ceramic parts of high resolution with sub-micrometer powder. Virtual Phys. Prototyp. 3, 3–11. 10.1080/17452750801907970

[B41] Fearing R. S. (1995). “Survey of sticking effects for micro parts handling,” in Proceedings 1995 IEEE/RSJ International Conference on Intelligent Robots and Systems. Human Robot Interaction and Cooperative Robots (IEEE) (IEEE), 212–217.

[B42] Feng K. Xu Q. Tam L. M. (2022). Design and development of a dexterous bilateral robotic microinjection system based on haptic feedback. IEEE Trans. Automation Sci. Eng., 1–11. 10.1109/tase.2022.3182409

[B43] Fox I. J. Daley G. Q. Goldman S. A. Huard J. Kamp T. J. Trucco M. (2014). Stem cell therapy. Use of differentiated pluripotent stem cells as replacement therapy for treating disease. Science 345, 1247391. 10.1126/science.1247391 25146295 PMC4329726

[B44] Fusco S. Sakar M. S. Kennedy S. Peters C. Pane S. Mooney D. (2014). “Self-folding mobile microrobots for biomedical applications. Robotics and Automation (ICRA),” in 2014 IEEE International Conference on (IEEE), 3777–3782.

[B45] Gao W. Dong R. Thamphiwatana S. Li J. Gao W. Zhang L. (2015). Artificial micromotors in the mouse’s stomach: A step toward *in vivo* use of synthetic motors. ACS Nano 9, 117–123. 10.1021/nn507097k 25549040 PMC4310033

[B46] Gao W. Manesh K. M. Hua J. Sattayasamitsathit S. Wang J. (2011). Hybrid nanomotor: A catalytically/magnetically powered adaptive nanowire swimmer. Small 7, 2047–2051. 10.1002/smll.201100213 21626685

[B47] Gao W. Wang J. (2014). Synthetic micro/nanomotors in drug delivery. Nanoscale 6, 10486–10494. 10.1039/c4nr03124e 25096021

[B48] Ghadiri R. Weigel T. Esen C. Ostendorf A. (2012). Microassembly of complex and three-dimensional microstructures using holographic optical tweezers. J. Micromechanics Microengineering 22, 065016. 10.1088/0960-1317/22/6/065016

[B49] Ghosh A. Yoon C. Ongaro F. Scheggi S. Selaru F. M. Misra S. (2017). Stimuli-responsive soft untethered grippers for drug delivery and robotic surgery. Front. Mech. Eng. 3, 7. 10.3389/fmech.2017.00007 31516892 PMC6740326

[B50] Gil B. Li B. Gao A. Yang G. Z. (2020). Miniaturized piezo force sensor for a medical catheter and implantable device. ACS Appl. Electron. Mater. 2, 2669–2677. 10.1021/acsaelm.0c00538 32879913 PMC7450887

[B51] Gorochowski T. E. Hauert S. Ju K. Marucci L. Stillman N. R. Tang T. Y. D. (2020). Toward engineering biosystems with emergent collective functions. Front. Bioeng. Biotechnol. 8, 705. 10.3389/fbioe.2020.00705 32671054 PMC7332988

[B52] Gorochowski T. E. Karr J. R. Parmeggiani F. Yordanov B. (2021). Editorial: Computer-aided biodesign across scales. Front. Bioeng. Biotechnol. 9, 700418. 10.3389/fbioe.2021.700418 34211968 PMC8240955

[B53] Grammatikopoulou M. Yang G. Z. (2019). Three-dimensional pose estimation of optically transparent microrobots. IEEE Robotics Automation Lett. 5, 72–79. 10.1109/lra.2019.2942272

[B54] Greco F. V. Tarnowski M. J. Gorochowski T. E. (2019). Living computers powered by biochemistry. Biochem. 41, 14–18. 10.1042/BIO04103014

[B55] Grexa I. Fekete T. Molnár J. Molnár K. Vizsnyiczai G. Ormos P. (2020). Single-cell elasticity measurement with an optically actuated microrobot. Micromachines 11, 882. 10.3390/mi11090882 32972024 PMC7570390

[B56] Grier D. G. (2003). A revolution in optical manipulation. nature 424, 810–816. 10.1038/nature01935 12917694

[B57] Grilli S. Ferraro P. (2008). Dielectrophoretic trapping of suspended particles by selective pyroelectric effect in lithium niobate crystals. Appl. Phys. Lett. 92, 232902. 10.1063/1.2943319

[B58] Grutzeck H. (2005). Investigations of the capillary effect for gripping silicon chips. Microsyst. Technol. 11, 194–203. 10.1007/s00542-004-0467-3

[B59] Guix M. Meyer A. K. Koch B. Schmidt O. G. (2016). Carbonate-based janus micromotors moving in ultra-light acidic environment generated by hela cells *in situ* . Sci. Rep. 6, 21701–21707. 10.1038/srep21701 26905939 PMC4764847

[B60] Gultepe E. Randhawa J. S. Kadam S. Yamanaka S. Selaru F. M. Shin E. J. (2013a). Biopsy with thermally-responsive untethered microtools. Adv. Mater. 25, 514–519. 10.1002/adma.201203348 23047708 PMC3832625

[B61] Gultepe E. Yamanaka S. Laflin K. E. Kadam S. Shim Y. Olaru A. V. (2013b). Biologic tissue sampling with untethered microgrippers. Gastroenterology 144, 691–693. 10.1053/j.gastro.2013.01.066 23399954 PMC3626272

[B62] Guo N. Leu M. C. (2013). Additive manufacturing: Technology, applications and research needs. Front. Mech. Eng. 8, 215–243. 10.1007/s11465-013-0248-8

[B63] Gyak K. W. Jeon S. Ha L. Kim S. Kim J. Lee K. S. (2019). Magnetically actuated sicn-based ceramic microrobot for guided cell delivery. Adv. Healthc. Mater. 8, 2000055. 10.1002/adhm.202000055 31596550

[B64] Hajba L. Guttman A. (2014). Circulating tumor-cell detection and capture using microfluidic devices. TrAC trends Anal. Chem. 59, 9–16. 10.1016/j.trac.2014.02.017

[B65] Haliyo D. S. Regnier S. Guinot J. C. (2003). [mü]MAD, the adhesion based dynamic micro-manipulator. Eur. J. Mech. 22, 903–916. 10.1016/s0997-7538(03)00071-8

[B66] Hanson L. Reinhardt W. Miskin M. (2022). Microrobot controlled electrodeposition metamaterial. Bulletin of the American Physical Society.

[B67] Hattori T. Kamiyama K. Kojima M. Horade M. (2015). “Generation of swirl flow by needle vibration for micro manipulation,” in Ieee/rsj International Conference on Intelligent Robots and Systems, 772–777.

[B68] Hauert S. Bhatia S. N. (2014). Mechanisms of cooperation in cancer nanomedicine: Towards systems nanotechnology. Trends Biotechnol. 32, 448–455. 10.1016/j.tibtech.2014.06.010 25086728 PMC4295824

[B69] Hosney A. Abdalla J. Amin I. S. Hamdi N. Khalil I. S. (2016). “ *In vitro* validation of clearing clogged vessels using microrobots,” in 2016 6th IEEE International Conference on Biomedical Robotics and Biomechatronics (BioRob) (IEEE) (IEEE), 272–277.

[B70] Iacovacci V. Ricotti L. Sinibaldi E. Signore G. Vistoli F. Menciassi A. (2018). An intravascular magnetic catheter enables the retrieval of nanoagents from the bloodstream. Adv. Sci. 5, 1800807. 10.1002/advs.201800807 PMC6145422 30250809

[B71] Ionov L. (2013). 3d microfabrication using stimuli-responsive self-folding polymer films. Polym. Rev. 53, 92–107. 10.1080/15583724.2012.751923

[B72] Jang D. Jeong J. Song H. Chung S. K. (2019). Targeted drug delivery technology using untethered microrobots: A review. J. Micromechanics Microengineering 29, 053002. 10.1088/1361-6439/ab087d

[B73] Jeong J. Jang D. Kim D. Lee D. Chung S. K. (2020). Acoustic bubble-based drug manipulation: Carrying, releasing and penetrating for targeted drug delivery using an electromagnetically actuated microrobot. Sensors Actuators A Phys. 306, 111973. 10.1016/j.sna.2020.111973

[B74] Jin Q. Yang Y. Jackson J. A. Yoon C. Gracias D. H. (2020). Untethered single cell grippers for active biopsy. Nano Lett. 20, 5383–5390. 10.1021/acs.nanolett.0c01729 32463679 PMC7405256

[B75] Jin Z. Zhang Z. Gu G. X. (2019). Autonomous *in-situ* correction of fused deposition modeling printers using computer vision and deep learning. Manuf. Lett. 22, 11–15. 10.1016/j.mfglet.2019.09.005

[B76] Jing W. Chowdhury S. Guix M. Wang J. An Z. Johnson B. V. (2018). A microforce-sensing mobile microrobot for automated micromanipulation tasks. IEEE Trans. Automation Sci. Eng. 16, 518–530. 10.1109/tase.2018.2833810

[B77] Justus K. B. Hellebrekers T. Lewis D. D. Wood A. Ingham C. Majidi C. (2019). A biosensing soft robot: Autonomous parsing of chemical signals through integrated organic and inorganic interfaces. Sci. Robotics 4, eaax0765. 10.1126/scirobotics.aax0765 33137770

[B78] Kagan D. Benchimol M. J. Claussen J. C. Chuluun-Erdene E. Esener S. Wang J. (2012). Acoustic droplet vaporization and propulsion of perfluorocarbon-loaded microbullets for targeted tissue penetration and deformation. Angew. Chem. Int. Ed. 51, 7519–7522. 10.1002/anie.201201902 PMC3477603 22692791

[B79] Karshalev E. Chen C. Marolt G. Martín A. Campos I. Castillo R. (2017). Utilizing iron’s attractive chemical and magnetic properties in microrocket design, extended motion, and unique performance. Small 13, 1700035. 10.1002/smll.201700035 28394480

[B80] Kawahara T. Sugita M. Hagiwara M. Arai F. Kawano H. Shihira-Ishikawa I. (2013). On-chip microrobot for investigating the response of aquatic microorganisms to mechanical stimulation. Lab a Chip 13, 1070–1078. 10.1039/c2lc41190c 23314607

[B81] Khammash M. H. (2022). Cybergenetics: Theory and applications of genetic control systems. Proc. IEEE 110, 631–658. 10.1109/JPROC.2022.3170599

[B82] Khoo Z. X. Teoh J. E. M. Liu Y. Chua C. K. Yang S. An J. (2015). 3d printing of smart materials: A review on recent progresses in 4d printing. Virtual Phys. Prototyp. 10, 103–122. 10.1080/17452759.2015.1097054

[B83] Kim D. Park J. Kim K. Park H. H. Ahn S. (2015). “Propulsion and control of implantable micro-robot based on wireless power transfer,” in Wireless Power Transfer Conference, 1–4.

[B84] Kim D. H. Kim B. Shim J. H. (2005b). A superelastic alloy microgripper with embedded electromagnetic actuators and piezoelectric sensors. Proc. Spie 5604, 230–237.

[B85] Kim D. H. Lee M. G. Kim B. Sun Y. (2005a). A superelastic alloy microgripper with embedded electromagnetic actuators and piezoelectric force sensors: A numerical and experimental study. Smart Mater. Struct. 14, 1265–1272. 10.1088/0964-1726/14/6/019

[B86] Kim K. Liu X. Zhang Y. Sun Y. (2008). “Micronewton force-controlled manipulation of biomaterials using a monolithic mems microgripper with two-axis force feedback,” in IEEE International Conference on Robotics and Automation, 3100–3105.

[B87] Kim S. Qiu F. Kim S. Ghanbari A. Moon C. Zhang L. (2013). Fabrication and characterization of magnetic microrobots for three-dimensional cell culture and targeted transportation. Adv. Mater. 25, 5863–5868. 10.1002/adma.201301484 23864519 PMC4260689

[B88] Knoepfler P. S. (2015). From bench to fda to bedside: Us regulatory trends for new stem cell therapies. Adv. drug Deliv. Rev. 82, 192–196. 10.1016/j.addr.2014.12.001 25489841 PMC4398607

[B89] Kobayashi K. Yoon C. Oh S. H. Pagaduan J. V. Gracias D. H. (2018). Biodegradable thermomagnetically responsive soft untethered grippers. ACS Appl. Mater. interfaces 11, 151–159. 10.1021/acsami.8b15646 30525417

[B90] Kuo J. C. Tung S. W. Yang Y. J. (2014). A hydrogel-based intravascular microgripper manipulated using magnetic fields. Sensors Actuators A Phys. 211, 121–130. 10.1016/j.sna.2014.02.028

[B91] Lee J. H. Lee K. H. Chae J. B. Rhee K. Sang K. C. (2013). On-chip micromanipulation by ac-ewod driven twin bubbles. Sensors Actuators A Phys. 195, 167–174. 10.1016/j.sna.2012.07.019

[B92] Leong T. G. Randall C. L. Benson B. R. Bassik N. Stern G. M. Gracias D. H. (2009). Tetherless thermobiochemically actuated microgrippers. Proc. Natl. Acad. Sci. U. S. A. 106, 703–708. 10.1073/pnas.0807698106 19139411 PMC2630075

[B93] Li C. Guo C. Fitzpatrick V. Ibrahim A. Zwierstra M. J. Hanna P. (2020). Design of biodegradable, implantable devices towards clinical translation. Nat. Rev. Mater. 5, 61–81. 10.1038/s41578-019-0150-z

[B94] Li J. de Ávila B. E. F. Gao W. Zhang L. Wang J. (2017). Micro/nanorobots for biomedicine: Delivery, surgery, sensing, and detoxification. Sci. Robot. 2, eaam6431. 10.1126/scirobotics.aam6431 31552379 PMC6759331

[B95] Li J. Li X. Luo T. Wang R. Liu C. Chen S. (2018). Development of a magnetic microrobot for carrying and delivering targeted cells. Sci. Robotics 3, eaat8829. 10.1126/scirobotics.aat8829 33141689

[B96] Li J. Mayorga-Martinez C. C. Ohl C. D. Pumera M. (2022). Ultrasonically propelled micro-and nanorobots. Adv. Funct. Mater. 32, 2102265. 10.1002/adfm.202102265

[B97] Li J. Pumera M. (2021). 3d printing of functional microrobots. Chem. Soc. Rev. 50, 2794–2838. 10.1039/d0cs01062f 33470252

[B98] Lin Z. Jiang T. Shang J. (2021). The emerging technology of biohybrid micro-robots: A review. Bio-Design Manuf. 5, 107–132. 10.1007/s42242-021-00135-6

[B99] Liu D. Wang T. Lu Y. (2022). Untethered microrobots for active drug delivery: From rational design to clinical settings. Adv. Healthc. Mater. 11, 2102253. 10.1002/adhm.202102253 34767306

[B100] Liu J. Chen T. Liu H. Chou X. Sun L. (2015). “Pzt driven triple-finger end effectors for micro-manipulation,” in IEEE International Conference on Cyber Technology in Automation, Control, and Intelligent Systems (IEEE), 1156–1161.

[B101] Liu J. Zhou Y. X. Yu T. H. (2003). Freeze tweezer to manipulate mini/micro objects. J. Micromechanics Microengineering 14, 269–276. 10.1088/0960-1317/14/2/015

[B102] Lu H. Yang Y. Lin X. Shi P. Shen Y. (2019). Low-invasive cell injection: Low-invasive cell injection based on rotational microrobot (adv. Biosys. 7/2019). Adv. Biosyst. 3, 1970071. 10.1002/adbi.201970071 32648674

[B103] Lu Z. Moraes C. Ye G. Simmons C. A. Sun Y. (2010). Single cell deposition and patterning with a robotic system. PLoS One 5, e13542. 10.1371/journal.pone.0013542 21042403 PMC2958835

[B104] Lyu X. Liu X. Zhou C. Duan S. Xu P. Dai J. (2021). Active, yet little mobility: Asymmetric decomposition of h2o2 is not sufficient in propelling catalytic micromotors. J. Am. Chem. Soc. 143, 12154–12164. 10.1021/jacs.1c04501 34339185

[B105] Malachowski K. Breger J. Kwag H. R. Wang M. O. Fisher J. P. Selaru F. M. (2014). Stimuli-responsive theragrippers for chemomechanical controlled release. Angew. Chem. Int. Ed. 53, 8045–8049. 10.1002/anie.201311047 PMC4315180 24634136

[B106] Martella D. Nocentini S. Nuzhdin D. Parmeggiani C. Wiersma D. S. (2017). Photonic microhand with autonomous action. Adv. Mater. 29, 1704047. 10.1002/adma.201704047 28976033

[B107] Marucci L. Barberis M. Karr J. Ray O. Race P. R. de Souza Andrade M. (2020). Computer-aided whole-cell design: Taking a holistic approach by integrating synthetic with systems biology. Front. Bioeng. Biotechnol. 8, 942. 10.3389/fbioe.2020.00942 32850764 PMC7426639

[B108] Menciassi A. Eisinberg A. Carrozza M. C. Dario P. (2003). Force sensing microinstrument for measuring tissue properties and pulse in microsurgery. IEEE/ASME Trans. mechatronics 8, 10–17. 10.1109/tmech.2003.809153

[B109] Molhave K. Hansen O. (2005). Electro-thermally actuated microgrippers with integrated force-feedback. J. Micromechanics Microengineering 15, 1265–1270. 10.1088/0960-1317/15/6/018

[B110] Muiños-Landin S. Fischer A. Holubec V. Cichos F. (2021). Reinforcement learning with artificial microswimmers. Sci. Robotics 6, eabd9285. 10.1126/scirobotics.abd9285 34043550

[B111] Munasinghe K. C. Bowatta B. G. C. T. Abayarathne H. Y. R. Kumararathna N. Maduwantha L. K. A. H. Arachchige N. M. P. (2016). “New mems based micro gripper using sma for micro level object manipulation and assembling,” in Moratuwa Engineering Research Conference, 36–41.

[B112] Nah S. K. Zhong Z. W. (2007). A microgripper using piezoelectric actuation for micro-object manipulation. Sensors Actuators A Phys. 133, 218–224. 10.1016/j.sna.2006.03.014

[B113] Nelson B. J. Kaliakatsos I. K. Abbott J. J. (2010). Microrobots for minimally invasive medicine. Annu. Rev. Biomed. Eng. 12, 55–85. 10.1146/annurev-bioeng-010510-103409 20415589

[B114] Ongaro F. Pacchierotti C. Yoon C. Prattichizzo D. Gracias D. H. Misra S. (2016b). “Evaluation of an electromagnetic system with haptic feedback for control of untethered, soft grippers affected by disturbances,” in 2016 6th IEEE International Conference on Biomedical Robotics and Biomechatronics (BioRob) (IEEE) (IEEE), 900–905.

[B115] Ongaro F. Yoon C. van den Brink F. Abayazid M. Oh S. H. Gracias D. H. (2016a). “Control of untethered soft grippers for pick-and-place tasks. Biomedical Robotics and Biomechatronics (BioRob),” in 2016 6th IEEE International Conference on (IEEE) (IEEE), 299–304. 10.1109/BIOROB.2016.7523642 PMC6719306 31482040

[B116] Pané S. Puigmartí-Luis J. Bergeles C. Chen X. Z. Pellicer E. Sort J. (2019). Imaging technologies for biomedical micro-and nanoswimmers. Adv. Mater. Technol. 4, 1800575. 10.1002/admt.201800575

[B117] Patino T. Feiner-Gracia N. Arque X. Miguel-Lopez A. Jannasch A. Stumpp T. (2018). Influence of enzyme quantity and distribution on the self-propulsion of non-janus urease-powered micromotors. J. Am. Chem. Soc. 140, 7896–7903. 10.1021/jacs.8b03460 29786426

[B118] Pedone E. de Cesare I. Zamora-Chimal C. G. Haener D. Postiglione L. La Regina A. (2021). Cheetah: A computational toolkit for cybergenetic control. ACS Synth. Biol. 10, 979–989. 10.1021/acssynbio.0c00463 33904719

[B119] Peraza-Hernandez E. A. Hartl D. J. Malak R. J. Lagoudas D. C. (2014). Origami-inspired active structures: A synthesis and review. Smart Mater. Struct. 23, 094001. 10.1088/0964-1726/23/9/094001

[B120] Petrovic D. Popovic G. Chatzitheodoridis E. Medico O. D. (2002). Gripping tools for handling and assembly of microcomponents. Int. Conf. Microelectron. 1, 247–250.

[B121] Piriyanont B. Moheimani S. O. R. Bazaei A. (2013). “Design and control of a mems micro-gripper with integrated electro-thermal force sensor,” in Control Conference, 479–484.

[B122] Power M. Anastasova S. Shanel S. Yang G. Z. (2017). “Towards hybrid microrobots using ph-and photo-responsive hydrogels for cancer targeting and drug delivery,” in 2017 IEEE International Conference on Robotics and Automation (ICRA) (IEEE), 6002–6007.

[B123] Power M. Thompson A. J. Anastasova S. Yang G. Z. (2018). A monolithic force-sensitive 3d microgripper fabricated on the tip of an optical fiber using 2-photon polymerization. Small 14, 1703964. 10.1002/smll.201703964 29479810

[B124] Power M. Yang G. Z. (2015). “Direct laser written passive micromanipulator end-effector for compliant object manipulation. Intelligent Robots and Systems (IROS),” in 2015 IEEE/RSJ International Conference on (IEEE), 790–797.

[B125] Qu J. Zhang W. Jung A. Cruz S. D. Liu X. (2015). “A mems microgripper with two-axis actuators and force sensors for microscale mechanical characterization of soft materials,” in IEEE International Conference on Automation Science and Engineering.

[B126] Randhawa J. S. Leong T. G. Bassik N. Benson B. R. Jochmans M. T. Gracias D. H. (2008). Pick-and-place using chemically actuated microgrippers. J. Am. Chem. Soc. 130, 17238–17239. 10.1021/ja806961p 19053402

[B127] Rao K. J. Li F. Meng L. Zheng H. Cai F. Wang W. (2015). A force to be reckoned with: A review of synthetic microswimmers powered by ultrasound. Small 11, 2836–2846. 10.1002/smll.201403621 25851515

[B128] Ren L. Nama N. McNeill J. M. Soto F. Yan Z. Liu W. (2019). 3d steerable, acoustically powered microswimmers for single-particle manipulation. Sci. Adv. 5, eaax3084. 10.1126/sciadv.aax3084 31692692 PMC6814402

[B129] Rubio Denniss A. Gorochowski T. E. Hauert S. (2022a). An open platform for high-resolution light-based control of microscopic collectives. Adv. Intell. Syst. 4, 2200009. 10.1002/aisy.202200009

[B130] Rubio Denniss A. Gorochowski T. Mateu L. F. Hauert S. (2022b). “Q-learning for real time control of heterogeneous microagent collectives,” in ALIFE 2022: The 2022 Conference on Artificial Life, vol. ALIFE 2022: The 2022 Conference on Artificial Life. 10.1162/isal_a_00497

[B131] Rus D. Tolley M. T. (2015). Design, fabrication and control of soft robots. Nature 521, 467–475. 10.1038/nature14543 26017446

[B132] Schmidt C. K. Medina-Sánchez M. Edmondson R. J. Schmidt O. G. (2020). Engineering microrobots for targeted cancer therapies from a medical perspective. Nat. Commun. 11, 5618–18. 10.1038/s41467-020-19322-7 33154372 PMC7645678

[B133] Schuerle S. Soleimany A. P. Yeh T. Anand G. M. Häberli M. Fleming H. E. (2019). Synthetic and living micropropellers for convection-enhanced nanoparticle transport. Sci. Adv. 5, eaav4803. 10.1126/sciadv.aav4803 31032412 PMC6486269

[B134] Servant A. Qiu F. Mazza M. Kostarelos K. Nelson B. J. (2015). Controlled *in vivo* swimming of a swarm of bacteria-like microrobotic flagella. Adv. Mater. 27, 2981–2988. 10.1002/adma.201404444 25850420

[B135] Setti M. (2007). Microscale and nanoscale robotics systems: Caracteristics, state of the art, and grand challenges. IEEE Robot. Autom. Mag. 14, 53–60.

[B136] Shi M. Yeatman E. M. (2021). A comparative review of artificial muscles for microsystem applications. Microsystems Nanoeng. 7, 95–19. 10.1038/s41378-021-00323-5 PMC8611050 34858630

[B137] Sitti M. Ceylan H. Hu W. Giltinan J. Turan M. Yim S. (2015). Biomedical applications of untethered mobile milli/microrobots. Proc. IEEE 103, 205–224. 10.1109/jproc.2014.2385105 PMC5063027 27746484

[B138] Song X. Sun R. Wang R. Zhou K. Xie R. Lin J. (2022). Puffball-inspired microrobotic systems with robust payload, strong protection, and targeted locomotion for on-demand drug delivery. Adv. Mater. 34, 2204791. 10.1002/adma.202204791 PMC11475404 36066311

[B139] Soto F. Karshalev E. Zhang F. Esteban Fernandez de Avila B. Nourhani A. Wang J. (2021). Smart materials for microrobots. Chem. Rev. 122, 5365–5403. 10.1021/acs.chemrev.0c00999 33522238

[B140] Soto F. Kupor D. Lopez-Ramirez M. A. Wei F. Karshalev E. Tang S. (2020). Onion-like multifunctional microtrap vehicles for attraction–trapping–destruction of biological threats. Angew. Chem. 132, 3508–3513. 10.1002/ange.201913872 31863710

[B141] Sridhar V. Podjaski F. Kröger J. Jiménez-Solano A. Park B. W. Lotsch B. V. (2020). Carbon nitride-based light-driven microswimmers with intrinsic photocharging ability. Proc. Natl. Acad. Sci. 117, 24748–24756. 10.1073/pnas.2007362117 32958654 PMC7547284

[B142] Srivastava S. K. Medina-Sánchez M. Koch B. Schmidt O. G. (2016). Medibots: Dual-action biogenic microdaggers for single-cell surgery and drug release. Adv. Mater. 28, 832–837. 10.1002/adma.201504327 26619085

[B143] Stanton M. M. Park B. W. Vilela D. Bente K. Faivre D. Sitti M. (2017). Magnetotactic bacteria powered biohybrids target e. coli biofilms. ACS Nano 11, 9968–9978. 10.1021/acsnano.7b04128 28933815

[B144] Stillman N. R. Kovacevic M. Balaz I. Hauert S. (2020). *In silico* modelling of cancer nanomedicine, across scales and transport barriers. NPJ Comput. Mater. 6, 92–10. 10.1038/s41524-020-00366-8

[B145] Teh K. S. (2017). Additive direct-write microfabrication for mems: A review. Front. Mech. Eng. 12, 490–509. 10.1007/s11465-017-0484-4

[B146] Terzopoulou A. Wang X. Chen X. Z. Palacios-Corella M. Pujante C. Herrero-Martín J. (2020). Biodegradable metal–organic framework-based microrobots (mofbots). Adv. Healthc. Mater. 9, 2001031. 10.1002/adhm.202001031 32902185

[B147] Thanuja M. Anupama C. Ranganath S. H. (2018). Bioengineered cellular and cell membrane-derived vehicles for actively targeted drug delivery: So near and yet so far. Adv. drug Deliv. Rev. 132, 57–80. 10.1016/j.addr.2018.06.012 29935987

[B148] Tottori S. Zhang L. Qiu F. Krawczyk K. K. Franco-Obregón A. Nelson B. J. (2012). Micromachines: Magnetic helical micromachines: Fabrication, controlled swimming, and cargo transport (adv. mater. 6/2012). Adv. Mater. 24, 709. 10.1002/adma.201290025 22213276

[B149] Vasudev A. Zhe J. (2008). A capillary microgripper based on electrowetting. Appl. Phys. Lett. 93, 103503–103579. 10.1063/1.2978402

[B150] Vikram Singh A. Sitti M. (2016). Targeted drug delivery and imaging using mobile milli/microrobots: A promising future towards theranostic pharmaceutical design. Curr. Pharm. Des. 22, 1418–1428. 10.2174/1381612822666151210124326 26654436

[B151] Volland B. Heerlein H. Rangelow I. (2002). Electrostatically driven microgripper. Microelectron. Eng. 61, 1015–1023. 10.1016/s0167-9317(02)00461-6

[B152] Walle B. L. Gauthier M. Chaillet N. (2007). “Dynamic modelling of a submerged freeze microgripper using a thermal network,” in 2007 IEEE/ASME international conference on advanced intelligent mechatronics (IEEE), 1–6.

[B153] Walle B. L. Gauthier M. Chaillet N. (2010). “Dynamic modelling of a submerged freeze microgripper using a thermal network,” in Ieee/asme International Conference on Advanced Intelligent Mechatronics, 1–6.

[B154] Wang F. Liang C. Tian Y. Zhao X. Zhang D. (2016b). Design and control of a compliant microgripper with a large amplification ratio for high-speed micro manipulation. IEEE/ASME Trans. Mechatronics 21, 1262–1271. 10.1109/TMECH.2016.2523564

[B155] Wang H. Shi Q. Guo Y. Li Y. Sun T. Huang Q. (2016a). Contact assembly of cell-laden hollow microtubes through automated micromanipulator tip locating. J. Micromechanics Microengineering 27, 015013. 10.1088/0960-1317/27/1/015013

[B156] Wang J. Soto F. Liu S. Yin Q. Purcell E. Zeng Y. (2022). Volbots: Volvox microalgae-based robots for multimode precision imaging and therapy. Adv. Funct. Mater. 32, 2201800. 10.1002/adfm.202201800

[B157] Wang Q. Wu Z. Huang J. Du Z. Yue Y. Chen D. (2021). Integration of sensing and shape-deforming capabilities for a bioinspired soft robot. Compos. Part B Eng. 223, 109116. 10.1016/j.compositesb.2021.109116

[B158] Wang X. Qin X. H. Hu C. Terzopoulou A. Chen X. Z. Huang T. Y. (2018). 3d printed enzymatically biodegradable soft helical microswimmers. Adv. Funct. Mater. 28, 1804107. 10.1002/adfm.201804107

[B159] Wason J. D. Wen J. T. Gorman J. J. Dagalakis N. G. (2012). Automated multiprobe microassembly using vision feedback. IEEE Trans. Robotics 28, 1090–1103. 10.1109/tro.2012.2200991

[B160] Wehner M. Truby R. L. Fitzgerald D. J. Mosadegh B. Whitesides G. M. Lewis J. A. (2016). An integrated design and fabrication strategy for entirely soft, autonomous robots. nature 536, 451–455. 10.1038/nature19100 27558065

[B161] Wei Y. Xu Q. (2015). An overview of micro-force sensing techniques. Sensors Actuators A Phys. 234, 359–374. 10.1016/j.sna.2015.09.028

[B162] Wierzbicki R. Houston K. Heerlein H. Barth W. Debski T. Eisinberg A. (2006). Design and fabrication of an electrostatically driven microgripper for blood vessel manipulation. Microelectron. Eng. 83, 1651–1654. 10.1016/j.mee.2006.01.110

[B163] Wijewardhane N. Denniss A. R. Uppington M. Hauser H. Gorochowski T. E. Piddini E. (2022). “Long-term imaging and spatio-temporal control of living cells using light,” in 2022 International Conference on Manipulation, Automation and Robotics at Small Scales (Toronto, Canada: MARSS), 1–6. 10.1109/MARSS55884.2022.9870487

[B164] Wong K. V. Hernandez A. (2012). A review of additive manufacturing. Int. Sch. Res. notices, 2012.

[B165] Wood D. Wood D. Wood D. Wood D. (2014). Modelling and experimental verification of heat dissipation mechanisms in an su-8 electrothermal microgripper. Microelectron. Eng. 124, 90–93. 10.1016/j.mee.2014.06.002

[B166] Wu Z. Chen Y. Mukasa D. Pak O. S. Gao W. (2020). Medical micro/nanorobots in complex media. Chem. Soc. Rev. 49, 8088–8112. 10.1039/d0cs00309c 32596700

[B167] Wu Z. Troll J. Jeong H. H. Wei Q. Stang M. Ziemssen F. (2018). A swarm of slippery micropropellers penetrates the vitreous body of the eye. Sci. Adv. 4, eaat4388. 10.1126/sciadv.aat4388 30406201 PMC6214640

[B168] Xie H. Régnier S. (2011). Development of a flexible robotic system for multiscale applications of micro/nanoscale manipulation and assembly. IEEE ASME Trans. Mechatronics 16, 266–276. 10.1109/tmech.2010.2040483

[B169] Xu Q. (2015). Design, fabrication, and testing of an mems microgripper with dual-axis force sensor. IEEE Sensors J. 15, 6017–6026. 10.1109/jsen.2015.2453013

[B170] Yang S. Xu Q. (2017). A review on actuation and sensing techniques for mems-based microgrippers. J. Micro-Bio Robotics 13, 1–14. 10.1007/s12213-017-0098-2

[B171] Yang Y. Bevan M. A. Li B. (2020a). Efficient navigation of colloidal robots in an unknown environment via deep reinforcement learning. Adv. Intell. Syst. 2, 1900106. 10.1002/aisy.201900106

[B172] Yang Y. Bevan M. A. Li B. (2020b). Micro/nano motor navigation and localization via deep reinforcement learning. Adv. Theory Simulations 3, 2000034. 10.1002/adts.202000034

[B173] Yasa I. C. Ceylan H. Bozuyuk U. Wild A. M. Sitti M. (2020). Elucidating the interaction dynamics between microswimmer body and immune system for medical microrobots. Sci. Robotics 5, eaaz3867. 10.1126/scirobotics.aaz3867 33022620

[B174] Yim S. Gultepe E. Gracias D. H. Sitti M. (2014). Biopsy using a magnetic capsule endoscope carrying, releasing, and retrieving untethered microgrippers. IEEE Trans. Biomed. Eng. 61, 513–521. 10.1109/tbme.2013.2283369 24108454 PMC4023810

[B175] Zhan Z. Chen L. Duan H. Chen Y. He M. Wang Z. (2021). 3d printed ultra-fast photothermal responsive shape memory hydrogel for microrobots. Int. J. Extreme Manuf. 4, 015302. 10.1088/2631-7990/ac376b

[B176] Zhang D. Barbot A. Lo B. Yang G. Z. (2020d). Distributed force control for microrobot manipulation via planar multi-spot optical tweezer. Adv. Opt. Mater. 8, 2000543. 10.1002/adom.202000543

[B177] Zhang D. Barbot A. Seichepine F. Lo F. P. W. Bai W. Yang G. Z. (2022). Micro-object pose estimation with sim-to-real transfer learning using small dataset. Commun. Phys. 5, 80–11. 10.1038/s42005-022-00844-z

[B178] Zhang D. Chen J. Li W. Bautista Salinas D. Yang G. Z. (2020b). A microsurgical robot research platform for robot-assisted microsurgery research and training. Int. J. Comput. assisted radiology Surg. 15, 15–25. 10.1007/s11548-019-02074-1 PMC6949326 31605352

[B179] Zhang D. Lo F. P. W. Zheng J. Q. Bai W. Yang G. Z. Lo B. (2020a). Data-driven microscopic pose and depth estimation for optical microrobot manipulation. Acs Photonics 7, 3003–3014. 10.1021/acsphotonics.0c00997

[B180] Zhang D. Wu Z. Chen J. Gao A. Chen X. Li P. (2020c). Automatic microsurgical skill assessment based on cross-domain transfer learning. IEEE Robotics Automation Lett. 5, 4148–4155. 10.1109/lra.2020.2989075

[B181] Zhang D. (2021). Perception and manipulation of microrobots via optical tweezer.

[B182] Zhang J. Diller E. (2016). Tetherless mobile micrograsping using a magnetic elastic composite material. Smart Mater. Struct. 25, 11LT03. 10.1088/0964-1726/25/11/11lt03

[B183] Zhang J. Onaizah O. Middleton K. You L. Diller E. (2017). Reliable grasping of three-dimensional untethered mobile magnetic microgripper for autonomous pick-and-place. IEEE Robotics Automation Lett. 2, 835–840. 10.1109/lra.2017.2657879

[B184] Zhang L. Abbott J. J. Dong L. Kratochvil B. E. Bell D. Nelson B. J. (2009). Artificial bacterial flagella: Fabrication and magnetic control. Appl. Phys. Lett. 94 (6), 064107-1–064107-3. College Park, Maryland: American Institute of Physics. 10.1063/1.3079655

[B185] Zhang Y. Zhang L. Yang L. Vong C. I. Chan K. F. Wu W. K. (2019b). Real-time tracking of fluorescent magnetic spore–based microrobots for remote detection of c. diff toxins. Sci. Adv. 5, eaau9650. 10.1126/sciadv.aau9650 30746470 PMC6357761

[B186] Zhang Z. Wang X. Liu J. Dai C. Sun Y. (2019a). Robotic micromanipulation: Fundamentals and applications. Annu. Rev. Control, Robotics, Aut. Syst. 2, 181–203. 10.1146/annurev-control-053018-023755

[B187] Zhu Y. Bazaei A. Moheimani S. O. R. Yuce M. R. (2011). Design, modeling, and control of a micromachined nanopositioner with integrated electrothermal actuation and sensing. J. Microelectromechanical Syst. 20, 711–719. 10.1109/jmems.2011.2140358

